# Rapid identification of early renal damage in asymptomatic hyperuricemia patients based on urine Raman spectroscopy and bioinformatics analysis

**DOI:** 10.3389/fchem.2023.1045697

**Published:** 2023-01-25

**Authors:** Xiaodong Kong, Haoyue Liang, Wei An, Sheng Bai, Yuyang Miao, Junlian Qiang, Haoyu Wang, Yuan Zhou, Qiang Zhang

**Affiliations:** ^1^ Department of Geriatrics, Tianjin Medical University General Hospital, Tianjin Geriatrics Institute, Tianjin, China; ^2^ State Key Laboratory of Experimental Hematology, National Clinical Research Center for Blood Diseases, Haihe Laboratory of Cell Ecosystem, Institute of Hematology and Blood Diseases Hospital, Chinese Academy of Medical Sciences & Peking Union Medical College, Tianjin, China; ^3^ Department of Ultrasound, Xiangya Hospital Central South University, Changsha, Hunan, China; ^4^ Tianjin Medical University, Tianjin, China

**Keywords:** hyperuricemia, asymptomatic, chronic kidney disease, dysregulation of metabolic signals, Raman spectroscopy, bioinformatics

## Abstract

**Objective:** The issue of when to start treatment in patients with hyperuricemia (HUA) without gout and chronic kidney disease (CKD) is both important and controversial. In this study, Raman spectroscopy (RS) was used to analyze urine samples, and key genes expressed differentially CKD were identified using bioinformatics. The biological functions and regulatory pathways of these key genes were preliminarily analyzed, and the relationship between them as well as the heterogeneity of the urine components of HUA was evaluated. This study provides new ideas for the rapid evaluation of renal function in patients with HUA and CKD, while providing an important reference for the new treatment strategy of HUA disease.

**Methods:** A physically examined population in 2021 was recruited as the research subjects. There were 10 cases with normal blood uric acid level and 31 cases with asymptomatic HUA diagnosis. The general clinical data were collected and the urine samples were analyzed by Raman spectroscopy. An identification model was also established by using the multidimensional multivariate method of orthogonal partial least squares discriminant analysis (OPLS-DA) model for statistical analysis of the data, key genes associated with CKD were identified using the Gene Expression Omnibus (GEO) database, and key biological pathways associated with renal function damage in CKD patients with HUA were analyzed.

**Results:** The Raman spectra showed significant differences in the levels of uric acid (640 cm^−1^), urea, creatinine (1,608, 1,706 cm^−1^), proteins/amino acids (642, 828, 1,556, 1,585, 1,587, 1,596, 1,603, 1,615 cm^−1^), and ketone body (1,643 cm^−1^) (*p* < 0.05). The top 10 differentially expressed genes (DEGs) associated with CKD (ALB, MYC, IL10, FOS, TOP2A, PLG, REN, FGA, CCNA2, and BUB1) were identified. Compared with the differential peak positions analyzed by the OPLS-DA model, it was found that the peak positions of glutathione, tryptophan and tyrosine may be important markers for the diagnosis and progression of CKD.

**Conclusion:** The progression of CKD was related to the expression of the *ALB, MYC, IL10, PLG, REN, and FGA* genes. Patients with HUA may have abnormalities in glutathione, tryptophan, tyrosine, and energy metabolism. The application of Raman spectroscopy to analyze urine samples and interpret the heterogeneity of the internal environment of asymptomatic HUA patients can be combined with the OPLS-DA model to mine the massive clinical and biochemical examination information on HUA patients. The results can also provide a reference for identifying the right time for intervention for uric acid as well as assist the early detection of changes in the internal environment of the body. Finally, this approach provides a useful technical supplement for exploring a low-cost, rapid evaluation and improving the timeliness of screening. Precise intervention of abnormal signal levels of internal environment and energy metabolism may be a potential way to delay renal injury in patients with HUA.

## Introduction

Uric acid, an end product of purine, is generally produced from nucleic acids and purine compounds after a series of metabolic processes in the liver, and it is eventually discharged from the body through the kidneys. However, the process of uric acid excretion is complex as, after its filtration through the glomeruli, a series of reabsorption and re-secretion processes occur in the proximal renal tubules, the mechanisms of which are still not fully understood. Excessive concentrations of uric acid deposited in the kidneys and joints can not only lead to kidney stones and gout, but also cause cardiovascular diseases such as hypertension and atherosclerosis (Deb and Sakharkar, 2021; Shin and Lee, 2021). Consequently, although uric acid was once considered to be simply an end product of purine metabolism with no physiological value, new knowledge has led to the understanding that it is actually a major endogenous water-soluble antioxidant whose antioxidant effects are similar to those of vitamin C (Gong et al., 2022). Hence, increases in the body’s uric acid levels could reflect the body’s attempt to increase the levels of endogenous antioxidants to eliminate free radicals and prevent toxicity and DNA damage as well as lipid peroxidation.

Hyperuricemia (HUA) is a metabolic syndrome caused by abnormal purine metabolism, and its diagnostic standard is internationally defined in terms of blood uric acid levels (male >420 μmol/L (7 mg/dL), female >357 μmol/L (6 mg/dL)) (Khanna et al., 2012a; Khanna et al., 2012b), The incidence of asymptomatic HUA in patients with chronic kidney disease (CKD) has been increasing every year but even though hyperuricemia can be secondary to kidney disease, it can still further aggravate the latter’s development. For instance, higher blood uric acid levels can significantly increase the prevalence of CKD while significantly decreasing survival rates. In addition, HUA is a strong predictor of acute and chronic renal failure as well as adverse prognosis (Fritz et al., 2022; Kandav et al., 2022). However, even though imaging techniques or examination of urine, blood and renal pathologies are performed to evaluate renal functions, the onset of HUA usually escapes detection. In China, there is a large number of non-gout asymptomatic HUA patients with multiple cardiovascular risk factors or ischemic heart diseases. Yet, it is a matter of concern that opinions on how to treat asymptomatic HUA, whether there is a need to treat it and how to determine the treatment standard, still diverge between clinicians. Hence, this represents an issue which still needs to be addressed. At the same time, focusing on the early detection and effective prevention of renal damage resulting from HUA can be greatly important for improving the quality of life of the affected population.

In recent years, an increasing number of researchers have been using Raman spectroscopy (RS) technology to distinguish the biomarkers of healthy subjects from those of patients at different stages of CKD (Feng et al., 2019; Chen et al., 2020; Zong et al., 2021). This technique provides various advantages in terms of non-invasiveness, micro-fine resolution, small sample size, no requirements for chemical reagents, high automation, and relatively low cost to provide molecular information and images of high resolutions in real-time (Devitt et al., 2018), and as such, RS has great potential for the early diagnosis of HUA with CKD. In particular, when analyzing urine spectra, the labeled peak positions reflect changes in the main components of the urine samples (such as urea, creatinine, creatine, and ketone bodies), thus providing a rapid assessment of renal function in patients with HUA and CKD (Robertson et al., 2022). So far, progress has been made in monitoring the level of uric acid in blood and urine by using Raman spectroscopy (Alula et al., 2019; Hernandez et al., 2019; Wang et al., 2019), and this approach has provided a simplified process for determining the uric acid content without the need for sample pretreatment. However, no information is available on the compositional analysis of urine for asymptomatic HUA patients with CKD.

**FIGURE 1:**
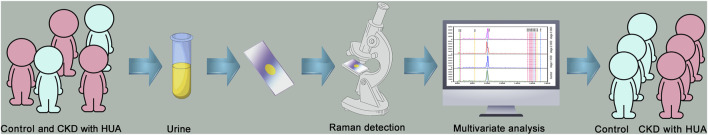
Schematic diagram for obtaining a Raman spectrum of urine: the spectrum for the subject under study was obtained by Raman spectroscopy. SIMCA 14.1 was used to analyze the data set through multivariate statistical analysis to distinguish patients with HUA and CKD.

For such patients, it has been speculated that there could be a specific energy supply and signal transduction processes that cause CKD. Thus, it was hypothesized that for HUA patients, there is a need to maintain a constant internal environment by relying on uric acid oxidation during the early stages of the disease (Liu et al., 2022). However, with disease progression, it becomes increasingly difficult for HUA patients to maintain the internal environment in a stable state. In this study, Raman spectroscopy, with an inelastic light scattering process, was used to capture the “fingerprint” of biomolecules in the urine of HUA patients, with the results expected to reflect changes in the expression of urea, creatinine, uric acid, protein, amino acids, ketones, and other substances in the *in vivo* microenvironment. Then, combined with gene chip data technology, the Bioinformation Analysis microarray data about CKD were downloaded and sorted from the GEO (Gene Expression Omnibus) database to find the key genes involved in the development of CKD. The combination of the relevant biological peak positions obtained by Raman spectroscopy with the molecular targets suggested by key genes opened a new way for analyzing information on the urine environment of CKD patients with HUA. It is expected that the results would assist in the clarification of the pathological mechanism of CKD caused by HUA (Figure 1).

## Methods

### Research subjects

The study participants were recruited between 28 September 2021, and 12 October 2021, from the physical examination center of the Healthcare Department (Department of Geriatrics) of the General Hospital of Tianjin Medical University, China. Based on the results of the physical examination, 10 subjects with normal levels of serum uric acid were randomly selected as the control group, and 31 with asymptomatic hyperuricemia were selected as the test group. Of these, 35 were males and six were females, with their ages varying between 35 and 97 years. The serum uric acid levels were 133–415 μmol/L and 424–595 μmol/L for the control and test groups, respectively. For the test group, the improved estimated glomerular filtration rate (eGFR) suitable for the Chinese population was calculated according to the CKD-EPI two-level race equation (Kong et al., 2013).

The specific formula is as follows:

$$eGFR\;\left(mL/\min/1.73m^{2}\right)=a^{*}\;{\left(Cr/\;b\right)}^{c_{*}}\;{\left(0.993\right)}^{age}$$

a: if female a = 144; if male a = 141
b: if female b = 0.7; if male b = 0.9
c: if female and Cr ≤ 0.7 mg/dl, c = −0.329, if female and Cr > 0.7 mg/dl, c = −1.209.

If male and Cr ≤ 0.7 mg/dl, c = −0.411, if male and Cr > 0.7 mg/dl, c = −1.209.

The CKD-EPI formula is currently recognized as an effective measure of CKD, especially for patients with GRF <60 mL/(min·1.73 m^2^), as it has higher specificity and is more useful for the evaluation of patients with normal or near-normal renal function.

In addition, the subjects in the test group were diagnosed and staged according to the clinical practice guidelines of the kidney disease outcome quality initiatives (K/DOQI) (National Kidney Foundation, 2002). As a result, 15 patients were classified as having the stage 1 CKD, defined as an eGFR greater than or equal to 90 (ml/min/1.73 m^2^), while 10 subjects were considered to be in the stage 2 CKD, with an eGFR between 60–89 (ml/min/1.73 m^2^), and 6 cases with an eGFR less than 60 (ml/min/1.73 m^2^) as assigned to the stage 3 CKD subgroup.

Patients were excluded from the study if they were taking drugs that affected uric acid metabolism (such as aspirin), had a previous history of primary kidney disease (such as glomerulonephritis), had complications arising from acute kidney injury, had received any renal replacement therapy or had urinary tract stones, gouty arthritis, severe edema, pleural effusion, or ascites as well as ketoacidosis. For this study, approval was obtained from the ethics committee of the General Hospital of Tianjin Medical University (IRB2022-WZ-132). All participating healthy individuals and patients provided written informed consent.

### Collection of general clinical data

The heights and weights of all subjects were measured, and blood was collected after 10 h of fasting. The blood samples were centrifuged to obtain the serum. Clinical and biochemical indices in the serum were measured with an automatic biochemical analyzer, while fresh urine samples were taken to determine the levels of the relevant urinary indices, including the urinary microalbumin creatinine ratio (ACR), urinary creatinine (UCR), urinary microalbumin (m-alb), urine pH, and urine specific gravity (SG). Similarly, indices related to renal function such as uric acid (uric), urea (urea), and creatinine (crea) levels, were measured, together with other biochemical indicators, including low-density and high-density lipoprotein cholesterol (LDL-C and HDL-C), direct bilirubin (DBIL), total bilirubin (TBIL), triglycerides (TG), total protein (TP), globulin (GLO), glycosylated hemoglobin (HbA1c), alpha fetoprotein (AFP), alanine transaminase (ALT), carcinoembryonic antigen (CEA), albumin (ALB), glucose (Glu), and total cholesterol (TC). Routine blood indicators, including the mean corpuscular volume (MCV), red blood cell SD (RBC-SD), red blood cell CV (RBC-CV), red blood cell (RBC), hemoglobin (HGB), mean red blood cell hemoglobin content (MCH), mean red blood cell hemoglobin concentration (MCHC), hematocrit (HCT), lymphocyte absolute value (LYM#), lymphocyte percentage (LYMPH%), neutrophil absolute value (NEU#), neutrophil granulocyte (NEU), white blood cell (WBC), monocytes percentage (MON), monocytes absolute value (MON#), eosinophilic granulocyte percentage (EOS), eosinophilic granulocyte absolute value (EOS#), basophils percentage (BAS), basophils absolute value (BAS#), platelet large cell ratio (P-LCR), platelet crit (PCT), mean platelet volume (FL), platelet distribution width (PDW), and platelet count (PLT) were also measured.

### Raman spectroscopy

Urine samples were uniformly collected and stored at −18°C before analysis. Subjects were then selected for Raman spectral analysis according to their blood uric acid levels. The frozen urine samples were thawed at room temperature, and after adding 5 μL to a glass slide, measurements were taken with a confocal Raman spectrometer (XploRA Raman microscope). In this case, the objective lens was 40 times, a 40-mW output power was applied, and a 785-nm laser was selected as the excitation light. Samples were also fixed to the XYZ three-dimensional platform for shooting using a 40 × 0.75 NA Nikon lens, while the laser was received applied to a spot size of about 2 × 2 μm. The other selected parameters included a single integration time of 250 s and measurements in the range of 600–1,800 cm^−1^. For each group, measurements were taken at 5–10 different sites, with a resolution of 1 cm^−1^ and at the same time, the background noise was assessed by acquisition of the Raman spectrum of the quartz slide. Lastly, data processing, including smoothing, background removal, and baseline correction was performed with Labspec6 software. All spectra were normalized with reference to the 1,650 cm^−1^ Raman peak as the internal standard.

### Raman spectroscopy data analysis and discrimination model establishment

SIMCA14.1 software was used to conduct supervised orthogonal partial least squares discriminant analysis (OPLS-DA) on the Raman spectral data from the urine samples from the control group as well as from patients with hyperuricemia and different CKD stages. The goodness-of-fit parameters *R* ^2^ and Q^2^ were then used to evaluate the performance of the OPLS model. Under the zero hypothesis, the model was verified by randomly resampling 200 times with a random change of the Y matrix. In addition, potential biomarkers were identified from statistically significant Raman peak positions in the classification model using cluster and V + S analyses. Based on a comprehensive consideration of the correlation coefficient, loadings, distance from the center in the V + S diagram and other parameters, Origin software was used to process the relevant data. Peak positions of variable importance (VIP) > 1.0 were considered to have a significant impact on the model.

### Screening the expression of key genes associated with the development of CKD

#### Data source

Data from the NCBI GEO (Gene Expression Omnibus, GEO; http://www.ncbi.nlm.nih.gov/geo/) database were used, specifically, dataset GSE66494 (species: *Homo sapiens*). The data included data from kidney biopsy samples from 53 CKD patients together with kidney biopsy data from eight healthy controls, all of which included data on gene expression profiles. All samples were obtained using a GPL6480 Agilent-014850 Whole Human Genome Microarray 4 × 44K G4112F platform.

#### DEG analysis

GEO2R, an online tool of the GEO database, was used for analysis. GEO2R used the GEOquery and limma packages in R language to select DEGs associated with the development of CKD. In this case, *p* < 0.05 and |logFC|>2 were selected as thresholds for identifying the key genes.

#### Functional enrichment analysis of DEGs

Using the online tool DAVID (the Database for Annotation, Visualization and Integration Discovery; https://david.ncifcrf.gov/), Gene Ontology (GO) functional annotation as well as Kyoto Encyclopedia of Genes and Genomes (KEGG) pathway enrichment analyses were carried out for key genes. The GO functions included three categories, namely molecular function (MF), cellular component (CC), and biological process (BP).

#### Protein interaction network and key gene analysis

STRING 11.0 (https://sring-db.org/), a protein interaction database, was used to explore the relationships between the proteins encoded by the DEGs using a protein-protein interaction network (PPI). The PPI results were imported and edited in Cytoscape 3.6.1 before analyzing the node scores within the network using network topology property index degree centrality. In this case, higher node scores reflected a greater likelihood of the node being a key node. Genes corresponding to the top 10 proteins (degree. TOP10) of the PPI network nodes were considered as hub genes with high connectivity in the network.

### Statistical analysis

In the multi-dimensional calculation of OPLS-DA were statistically significant in one dimension, the peak positions with VIP value >1.0 obtained by the OPLS-DA model were processed using the IBM SPSS Statistics 26 statistical software package. Normally distributed data were expressed as means ± standard deviation. One-way ANOVA was used for the comparison of means between groups, the LSD method was used for the comparison between two groups with uniform square differences, and Tamhane’s T2 method was used for the comparison between two groups with uneven variance. Non-normally distributed data were expressed by M (Q1-Q3), and the Kruskal Wallis test was used for comparisons between groups, with statistical significance set at *p* < 0.05. For the validation of clinical data, the chi square test was used for comparison between frequency data groups, and GraphPad Prism nine was used for the drawing of statistical correlation graphs.

## Results

### Raman spectroscopy of urine in CKD patients and controls

**FIGURE 2:**
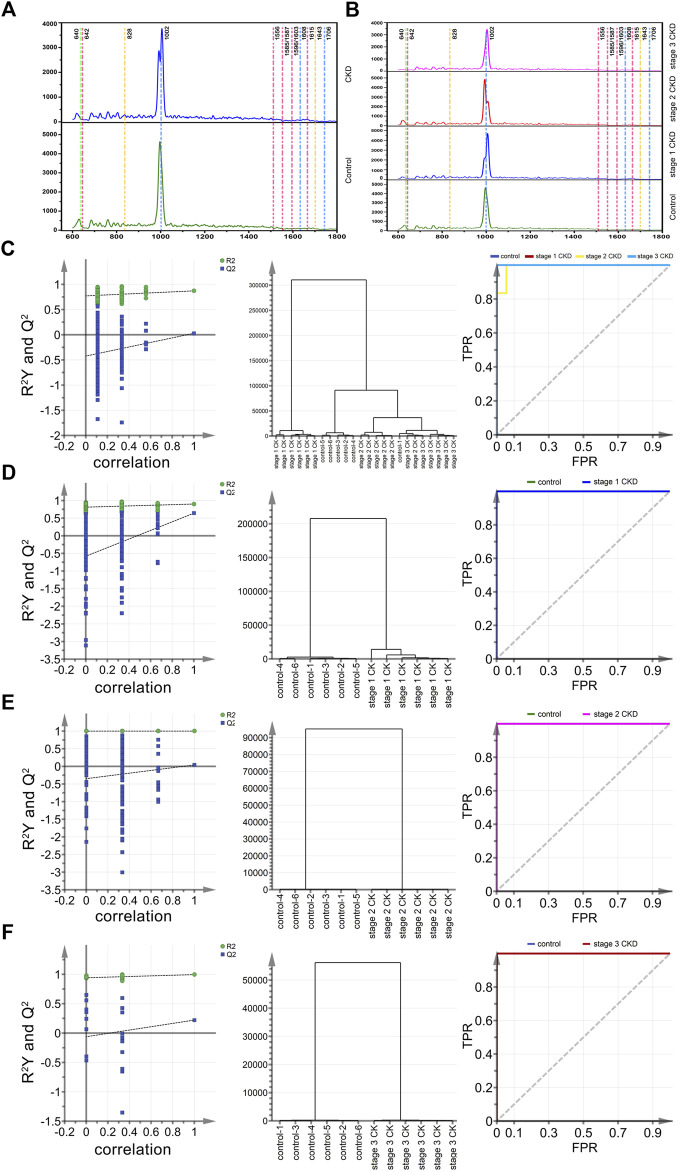
Mean spectral and multiparametric analysis of different CKD stages and control groups combined with HUA. **(A)** (from bottom to top) the average urine spectra of the control group (green solid line) and CKD subgroups (blue solid line). **(B)** (from bottom to top) the average urine spectra of the control group (green solid line), stage 1 CKD group (blue solid line), stage 2 CKD group (red solid line) and stage 3 CKD group (pink solid line), with the latter three combined with HUA; **(C)** The arrangement, clustering, and ROC curves of the control group, discriminated by OPLS and the CKD subgroup combined with HUA. **(D)** The arrangement, clustering and ROC diagram of OPLS discriminated control group and stage 1 CKD group combined with HUA; **(E)** The arrangement, clustering and ROC diagram of OPLS discriminated control group and stage 2 CKD group combined with HUA; **(F)** The arrangement, clustering and ROC diagram of OPLS discriminated control group and stage 3 CKD group combined with HUA.

In this study, a total of 258 Raman spectra of urine samples were obtained, and of these, 65 were for the control group, 93 were from stage 1 CKD of HUA patients, 62 were from stage 2 CKD of HUA patients and 38 were from stage 3 CKD of HUA patients. These results were presented in Figures 2A, B which show the Raman spectra of urine for the control group and the CKD group as well as for the control group and the CKD subgroups in the range of 600–1,800 cm^−1^. Supplementary Tables S1, S2 further highlight the assignment of peak positions which could the relevant in the Raman spectra of the urine. In Figure 2A, the green, pink, yellow, and blue dashed vertical lines in the figure indicate the peak positions related to uric acid (640 cm^−1^), protein, amino acid (642, 1,556, 1,585, 1,587, 1,596, 1,603, 1,615 cm^−1^), ketone bodies (828, 1,643 cm^−1^), urea and creatinine (1,608, 1,706 cm^−1^), respectively. Similarly, Figure 2B shows the Raman spectra of stage 1 up to stage 3 CKD, combined with HUA. The spectra had similar shapes, with Supplementary Figures S1, S2 also showing the standard deviation of the average spectrum of CKD multi samples. The results also showed that the standard deviation of this analysis method was low, which may reflect the actual situation of the samples.

However, it was often difficult to distinguish between the urine components of CKD patients and the controls based only on the above spectral patterns and peak positions. Hence, it is necessary to further identify peak positions that could assist in differentiating between the control and CKD subgroups so that, along with the OPLS-DA method and the classification model established by statistical analysis, the peaks can act as potential biomarkers.

### Preliminary screening and identification of potential biomarkers of HUA, combined with CKD using OPLS-DA model

Twenty-four characteristic spectra (six in each category) were randomly selected from the four sets of urine Raman spectra, namely, those of the control group, stage 1 CKD with HUA, stage 2 CKD with HUA, and stage 3 CKD with HUA. The sample data were analyzed and compared by applying the supervised OPLS-DA. Permutation analysis showed that the intercept of Q^2^ on the *y*-axis was negative, indicating that the OPLS-DA model was reliable and not an over-fit (Figures 2C–F). Under the OPLS-DA model, cluster analysis distinguished the Raman spectra of urine samples of the control group (five correct and one incorrect) from the CKD subgroups (six correct and 0 incorrect for stage 1 CKD combined with HUA, five correct and one incorrect for stage 2 CKD combined with HUA and six correct and 0 incorrect for stage 3 CKD combined with HUA) with 91.7% accuracy. Receiver operating characteristic (ROC) curves further reflected the high accuracy of discriminant analysis results (Figures 2C–F). The closer the area under the curve (AUC) is to 1, the higher is the authenticity of the identification method. The results of the pairwise combination model of the control group and the CKD subgroups showed that the model was not overfitted. Similarly, under the OPLS-DA model, cluster analysis helped to distinguish between the Raman spectra of urine samples of the control group and those who were at the different stages of CKD with 100% accuracy. The ROC curves indicated that the discriminant analysis results were highly accurate (Figures 2C–F).

**FIGURE 3:**
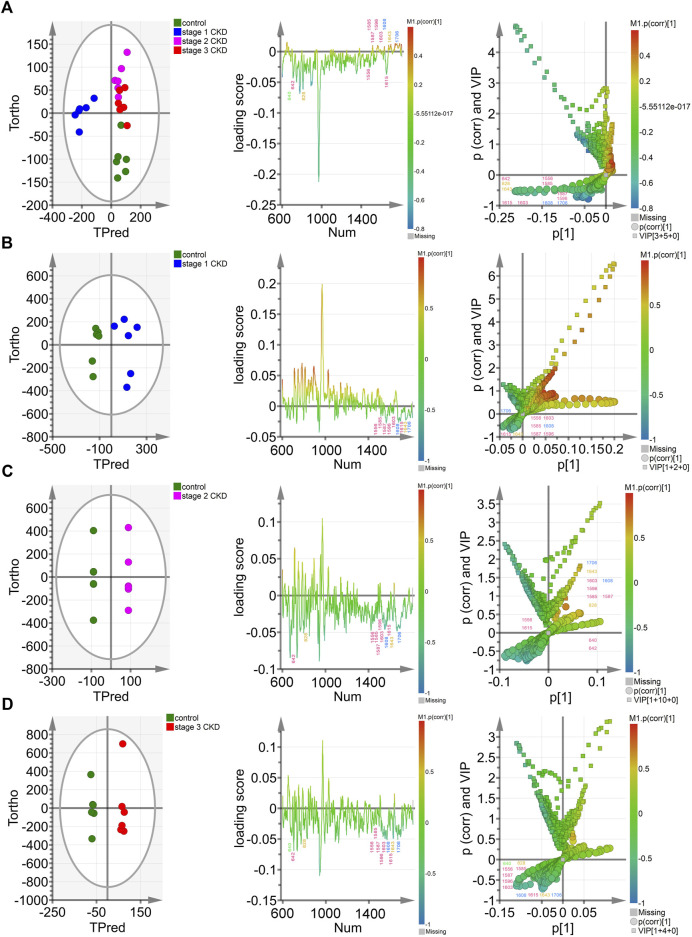
Multivariate analysis of control groups and different CKD stages with hyperuricemia **(A)** The OPLS-DA model was constructed between the control group and the three CKD one at different stages, and the Hotelling’s T2 ellipse score map, load map, and V + S map of the 95% confidence zone were drawn; **(B)**. The OPLS-DA model was constructed between the control group and stage 1 CKD group, before drawing the Hotelling’s T2 ellipse score map, load map, and V + S map of the 95% confidence zone; **(C)**. The OPLS-DA model was constructed in the control group and phase two CKD group, with the Hotelling’s T2 ellipse score map, load map and V + S map of the 95% confidence zone subsequently drawn; **(D)**. The OPLS-DA model was constructed between the control group and the stage 3 CKD group before drawing the Hotelling’s T2 ellipse score map, load map and V + S map of the 95% confidence zone.

The OPLS-DA score chart (the left column of Figure 3A) indicates that the four groups of samples were obviously clustered. In particular, the control group, the stage 2 CKD with HUA, and the stage 3 CKD with HUA groups were located on the positive half of the *x*-axis. On the other hand, the stage 1 CKD with HUA group was located on the negative half of the *x*-axis. At the same time, while the control group was found on the negative half of the *y*-axis, the stage 2 CKD with HUA group as well as the stage 3 CKD with HUA group were located on its positive half. These results reflected the differences between the control group and the CKD subgroups, allowing them to be clearly distinguished. Thus, it was established that OPLS-DA could distinguish between the spectral data of urine from the four groups, thereby providing conditions for analyzing their material composition. In the OPLS-DA score map (the left column of Figures 3B–D), the scattered points of the control group were located on the negative half of the *x*-axis, while the scattered points of the CKD subgroup were located on its positive half. The sample clustering was obvious and indicated that the model successfully discriminates and classifies the samples.

The OPLS-DA loading map (the left two columns of Figures 3A–D) confirmed the Raman peak positions that contributed to the identification of the control and CKD subgroups. The pink, blue, yellow, and green peak positions in the figure were related to protein/amino acid (642, 1,556, 1,585, 1,587, 1,596, 1,603, and 1,615 cm^−1^), urea/creatinine (1,608 and 1,706 cm^−1^), ketone body (828 and 1,643 cm^−1^), and uric acid (640 cm^−1^), respectively. These characteristic peak positions played a key role in the identification of the four groups of samples.

The OPLS-DA V + S diagram (the left three columns of Figures 3A–D) provides the main basis for identifying potential biomarkers in the control group and CKD subgroup models. The Raman peak positions of the selected models were differentiated, and their significance determined. The peak positions with VIP value >1.0 were considered potential biomarkers for the effective differentiation between the control group and the CKD subgroups. The 828-cm^−1^ peak position representing ketone body/protein/amino acid and the characteristic peak position of protein/amino acid (1,556, 1,585, 1,587 cm^−1^) were the most influential and biologically significant peaks in the model and played an important role in the identification of CKD samples, reflecting that the urine ketone bodies and some protein/amino acid contents of non-hyperuricemic people are higher than those of patients with CKD combined with HUA (Figures 3A–D).

### Statistical analysis of CKD with HUA and control groups

The peak positions obtained after the initial screening of the OPLS-DA model were verified by statistical analysis, and key peaks for identifying HUA-related renal injury were clearly identified. With reference to previous literature (Moreira et al., 2017; González-Solís et al., 2018; Moreira et al., 2018; Chen et al., 2020; Lin et al., 2020), it was confirmed that the 640-cm^−1^ peak represented uric acid, while the peaks at 642, 828, 1,556, 1,585, 1,587, 1,596, 1,603, and 1,615 cm^−1^ represented the peak positions of proteins, tyrosine, glutathione, tryptophan, carbene linkages, adenine, serine, and phenylalanine, respectively. Similarly, urea and creatinine were represented by the peaks at 1,608 and 1,706 cm^−1^, respectively, while the 828-cm^−1^ peak represents the peak position of glutathione/tryptophan/β-hydroxybutyric acid and the 1,643-cm^−1^ peak was indicative of β-hydroxybutyric acid. Supplementary Table S2 showed the detailed classification of the biomarkers.

There were no significant differences in the intensities of the 640, 642, 828, 1,556, 1,585, 1,587, 1,596, 1,603, 1,608, 1,615, and 1,706-cm^−1^ peaks between the urine of the three groups of CKD patients (*p* > 0.05). However, there was a significant difference in the peak at 1,643 cm^−1^ in the urine of stage 1 and stage 2 CKD patients (*p* = 0.007), and significant differences were also observed when comparing stage 1 CKD patients with HUA with those in stage 3 CKD (*p* = 0.001). Lastly, no significant differences between stage 2 CKD patients with HUA and stage 3 CKD patients (*p* > 0.05) were noted.

**FIGURE 4:**
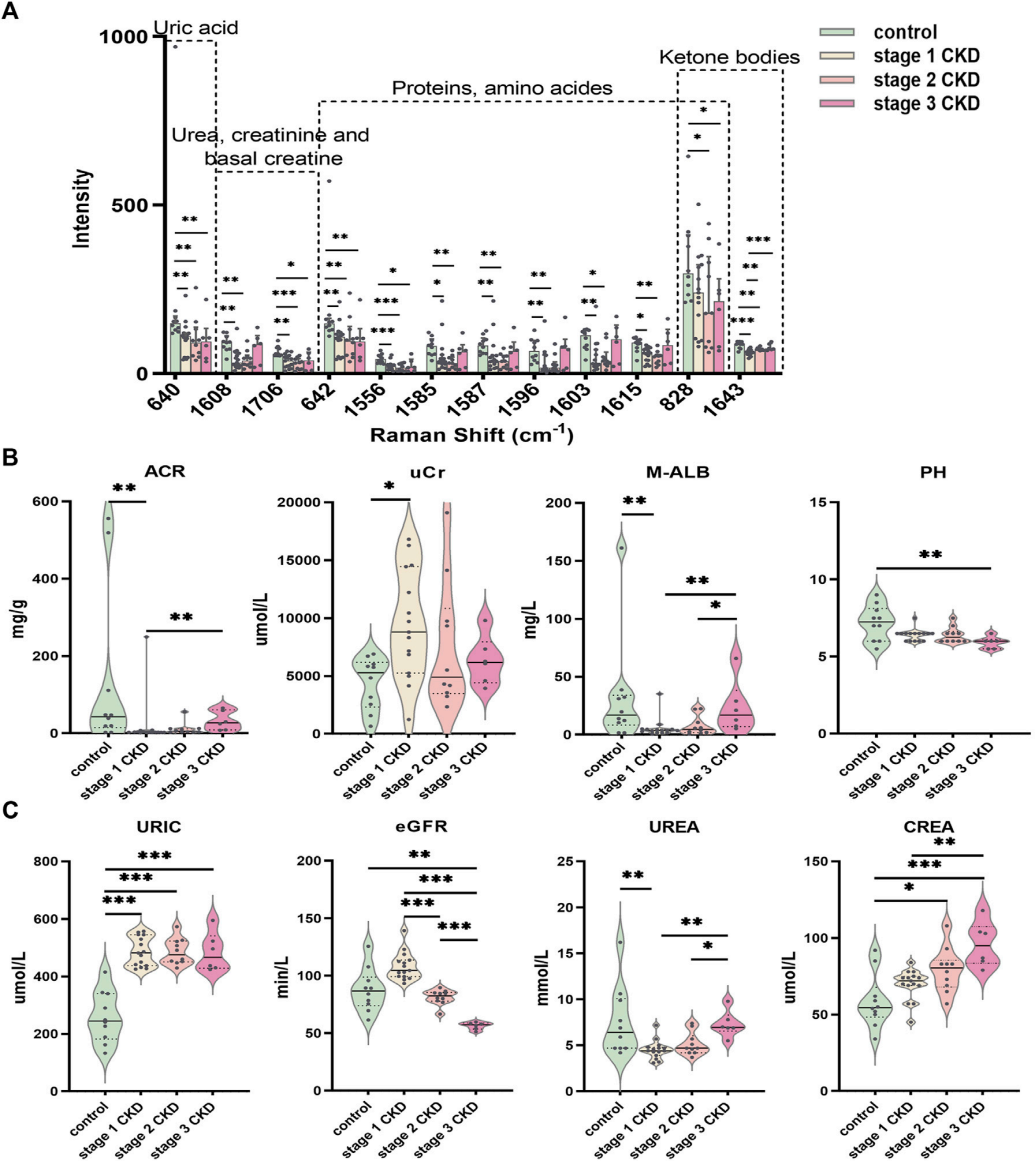
Statistically significant Raman spectral peak maps and evaluation of identified clinical indicators of renal function **(A)** Statistical analysis showed that the intensity of the uric acid peak, urea, creatinine, proteins/amino acids, adenine/serine, and ketone bodies in the urine of the CKD subgroup and control group; a Normally distributed data are expressed as mean ± standard deviation, and non-normally distributed data are expressed as median and interquartile range. **(B)** The piano chart of urine renal function evaluation indexes (ACR, UCR, M-ALB, pH) in different stages of CKD with HUA and the control group; **(C)** The piano diagram of the comparison of different CKD stages with HUA and the evaluation indexes of peripheral blood renal function (uric, eGFR, urea, crea) in the control group; The solid line in the piano chart is the median, and the dotted line is the upper and lower quartiles, *p < 0.05, **p < 0.01, ***p < 0.001.

The peak intensities of uric acid (640 cm^−1^), urea/creatinine (1,608, 1,706 cm^−1^), proteins/amino acids (642, 1,556, 1,585, 1,587, 1,596, 1,603, and 1,615 cm^−1^), and ketone bodies (1,643 cm^−1^) in stage 1 CKD patients with HUA were significantly different from those of control patients (*p* < 0.05). Likewise, the peak intensities of uric acid (640 cm^−1^), urea/creatinine (1,608, 1,706 cm^−1^), proteins/amino acids (642, 828, 1,556, 1,585, 1,587, 1,596, 1,603, and 1,615 cm^−1^), and ketone bodies (828, 1,643 cm^−1^) differed significantly between patients with stage 2 CKD and HUA and the controls (*p* < 0.05). Compared with the control group, the peak intensities of uric acid (640 cm^−1^), urea/creatinine (1,706 cm^−1^), proteins/amino acids (642, 828, and 1,556 cm^−1^), and ketone bodies (828 cm^−1^) in patients with stage 3 CKD patients and HUA were also statistically significant (*p* < 0.05). See Figure 4A and Supplementary Tables S1, S2.

Comparisons of the peripheral blood indices of renal function showed that the blood uric acid level for the control group was 259.0 ± 88.6 μmol L (range 133–415), while that of the stage 1 CKD group with HUA was 486.3 ± 50.3 μmol/L (range 425–557). Similarly, the blood uric acid level in the stage 2 CKD group with HUA was 486.0 ± 45.5 μmol/L (range 429–573), and the blood uric acid level in the stage 3 CKD group with HUA was 484.2 ± 67.4 μmol/L (range 424–595). Compared with the control, the serum uric acid levels were significantly higher in patients in the different CKD stages (*p* = 0.000).

The blood urea, urine microalbumin:creatinine ratio, and urine microalbumin levels in stage 1 CKD patients with HUA were lower those for the control (*p* = 0.005 and *p* = 0.008, respectively), and the creatinine levels in the urine were also higher than those in the control group (*p* = 0.010). The eGFR, blood creatinine, and urine pH level were not significantly different (*p* > 0.05), although stage 2 CKD patients with HUA had higher serum creatinine levels compared with the control group (*p* = 0.013). At the same time, there was no significant differences in the eGFR, urea, urine microalbumin creatinine ratio, urine creatinine, urine microalbumin, and urine pH levels (*p* > 0.05). The levels of eGFR and creatinine in stage 3 CKD patients with HUA were significantly higher than those in the control group (*p* = 0.003 and *p* = 0.000, respectively), and the urine pH was significantly lower than that in the control group (*p* = 0.002). There were no significant differences in blood urea, urine microalbumin creatinine ratio, urine creatinine, and urine microalbumin (*p* > 0.05).

A significantly higher eGFR was noted for stage 1 CKD patients with HUA compared with those in stage 2 (*p* = 0.000), but no significant differences were observed in terms of the serum uric acid, urea, serum creatinine, urine microalbumin creatinine ratio, urine creatinine, urine microalbumin, and urine pH (*p* > 0.05). The eGFR of stage 1 CKD patients with HUA was also higher compared with stage 3 CKD (*p* = 0.000), and in this case, the blood urea, blood creatinine, urine microalbumin creatinine ratio, and urine microalbumin were significantly lower compared with stage 3 CKD (*p* = 0.002, *p* = 0.003, *p* = 0.004, and *p* = 0.008, respectively), although no significant difference were present in blood uric acid, urine creatinine, and urine pH (*p* > 0.05). Finally, comparing the eGFR of stage 2 and stage 3 CKD patients with HUA indicated that the eGFR values were higher in the stage 2 patients (*p* = 0.000). At the same time, the blood urea and urine microalbumin were lower than those of stage 3 CKD patients (*p* = 0.030 and *p* = 0.046, respectively). There was no significant difference in blood creatinine, urine microalbumin: creatinine ratio, urine creatinine, and urine pH (*p* > 0.05). See Figures 4B, C and Supplementary Tables S3, S4.

**FIGURE 5:**
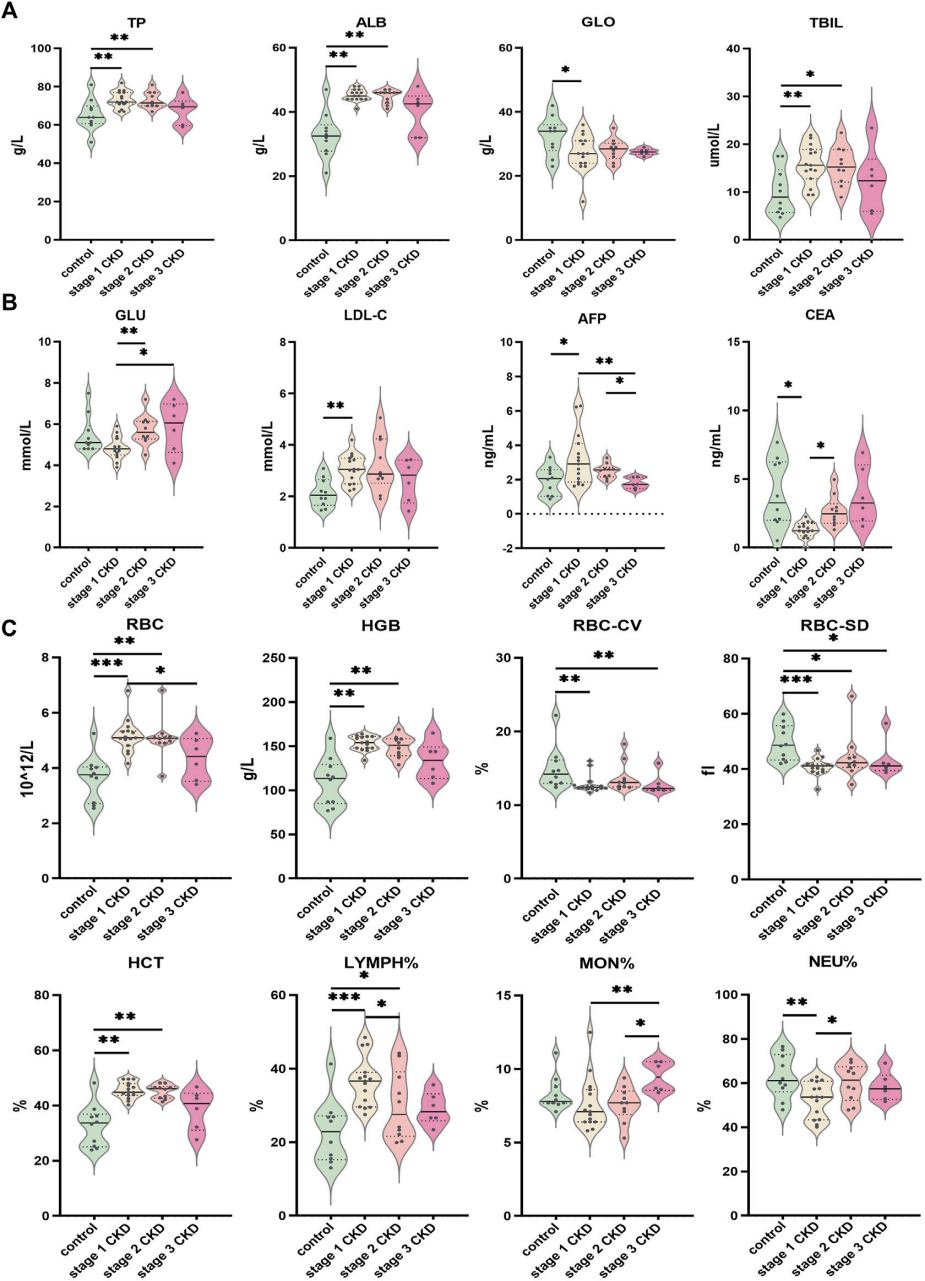
Comparison of clinical data of patients with different CKD stages with hyperuricemia and control group **(A)** Indices of liver function (TP, ALB, GLO, TBIL) for CKD at different stages; **(B)** Comparison of glucose and lipid metabolism (GLU, LDL-C) and tumor markers (AFP, CEA) in peripheral blood of different CKD stages combined with HUA and control group; **(C)** Comparison of peripheral blood routine indices (RBC, HGB, RBC-CV, RBC-SD, HCT, LYMPH%, MON%, NEU%) in different stages of CKD, combined with HUA and in the control group. The solid line in the piano chart is the median, and the dotted line is the upper and lower quartiles, *p < 0.05, **p < 0.01, ***p < 0.001.

When compared with other clinical data, age, sex, total protein, albumin, globulin, total bilirubin, glucose, total cholesterol, low-density lipoprotein, alpha fetoprotein, carcinoembryonic antigen, red blood cell count, hemoglobin, hematocrit, number of variation lines of red blood cell distribution width, standard deviation of red blood cell distribution width, percentage of neutrophils, absolute value of neutrophils, percentage of lymphocytes and percentage of monocytes, significant between-group or within-groups difference in the absolute values of monocytes and eosinophils were observed (*p* < 0.05). (See Figures 5A–C and Supplementary Tables S3, S4). In terms of the clinical data, BMI, urine specific gravity, glutamic pyruvic transaminase, direct bilirubin, glycosylated hemoglobin, triglycerides, high-density lipoprotein, average red blood cell volume, average red blood cell hemoglobin concentration, average red blood cell hemoglobin content, white blood cell count, lymphocyte absolute value, basophil percentage, basophil absolute value, platelet count, platelet volume distribution width, platelet average volume There was no significant difference in the platelet specific volume and large platelet ratio between or within the groups (*p* > 0.05). See Supplementary Tables S3, S4.

### Bioinformatics analysis of key DEGs in the development of CKD

#### Differentially expressed genes

**FIGURE 6:**
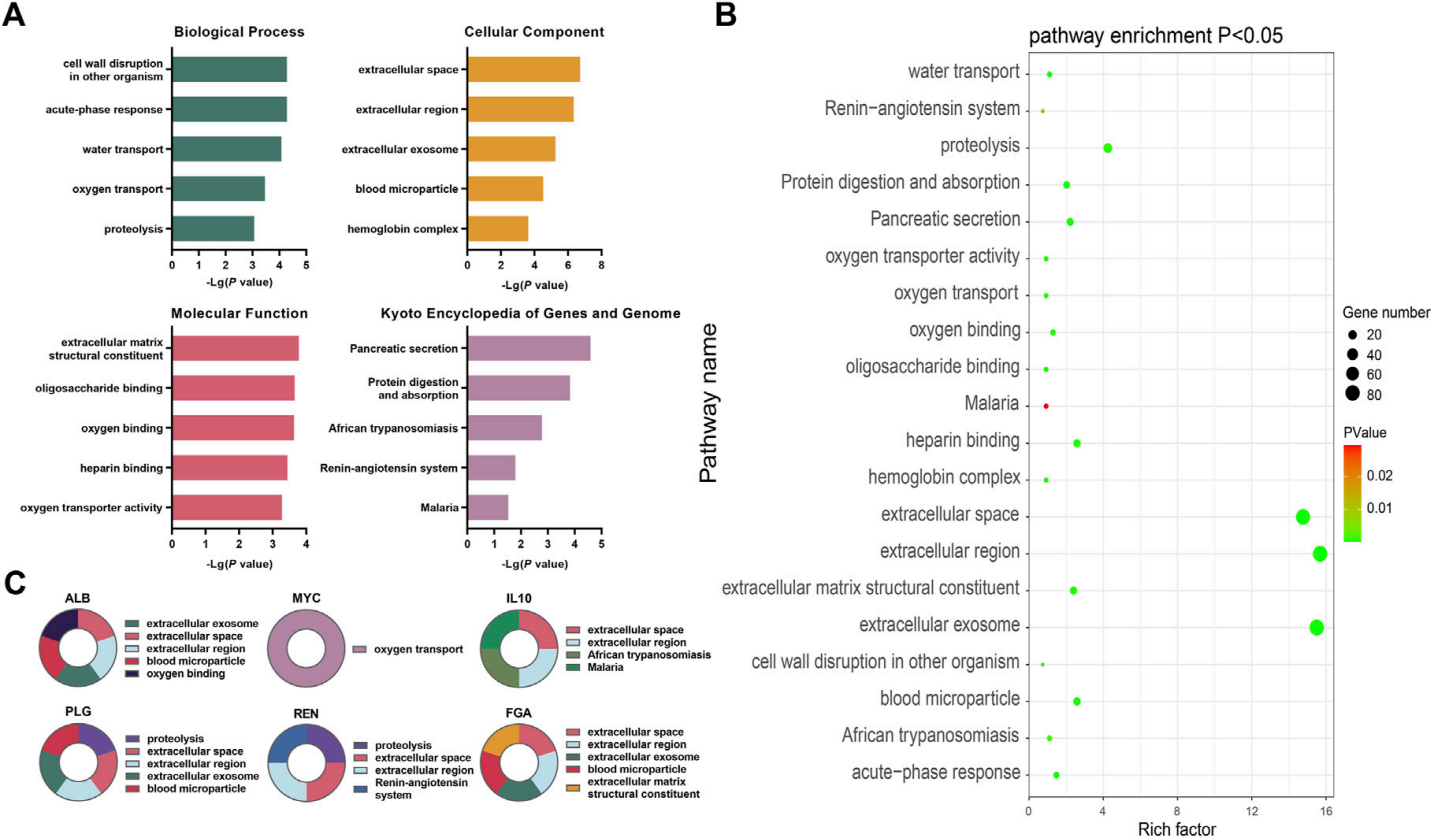
Bioinformatics analysis of key differentially expressed genes during the occurrence and development of CKD **(A)** The top five annotations in biological process, cell composition, molecular function, and KEGG pathway enrichment analysis of DEGs between the control group and the HUA with CKD subgroups; **(B)** Screening the top 10 CKD differential genes of PPI network degree value, combining with go functional annotation and KEGG pathway analysis, it was found that the key DEGs for the occurrence and development of CKD might be *ALB, MYC, IL10, PLG, REN* and *FGA*, and mapping the function and regulatory signal pathway of genes; **(C)** The bubble diagram of enrichment analysis between the control group and the HUA combined CKD subgroup shows that the larger the bubble, the more genes enriched in this functional pathway, and the closer the color of the bubble to green, indicating the greater significance.

A total of 581 DEGs were identified from the GSE66494 chip data, of which 432 were significantly upregulated (Supplementary Table S5) and 149 were significantly downregulated (Supplementary Table S6). The red or green dots in the figure represent the genes that were significantly upregulated or downregulated, respectively (Figure 6C).

#### GO and KEGG analyses of common DEGs using DAVID

The DEGs were found to be significantly enriched in 16 KEGG pathways, while the results for the GO categories included 103 genes in GO-BP, 35 in GO-CC, and 43 in GO-MF. Figure 6A shows the top five enriched GO functions after ordering the results from small to large *p*-values. Combined with the identification of the top 10 genes, the main enriched pathways in biological process (BP) were oxygen transport and protein hydrolysis. In terms of cell components (CC), extracellular space, extracellular region, exosomes, and blood microparticles were mostly enriched, while for molecular functions (MF), the DEGs were mainly involved in functions associated with extracellular matrix structural components and oxygen junctions. The KEGG analysis showed significant enrichment in pathways associated with African trypanosomiasis signaling pathway, renin angiotensin system signaling pathway, and the malaria signaling pathway (Figure 6A, Supplementary Tables S7–S10).

#### PPI network and identification of key candidate genes

The PPI network contained 376 nodes and 2044 interaction pairs. The topology score was high and could be regarded as the key node of the network. The hub genes were confirmed through the CytoHubba plug-in. The results showed that the hub genes are ALB, MYC, IL10, FOS, TOP2A, PLG, REN, FGA, CCNA2, and BUB1. The data suggested the presence of strong interactions between them. The GO functional analysis suggested that, of the top 10 genes, MYC is involved in oxygen transport while PLG and REN are involved in proteolysis. ALB, IL10, PLG, REN, and FGA were predicted to be located in the extracellular space and extracellular region, while the ALB, PLG, and FGA were predicted to be associated with exosomes and blood microparticles. FGA is a structural component of the extracellular matrix, while ALB is involved in oxygen binding; IL10 in African trypanosomiasis signaling and the malaria signaling pathway, and REN in the renin angiotensin system signaling pathway (see Figures 6A, B, Supplementary Figure S3 and Supplementary Tables S7–S10 for details).

The intensity values of the top 10 genes were derived from the data of 53 CKD populations and eight normal populations in the database. The specific gene expression values are shown in Supplementary Figure S3. The upregulated genes were ALB, IL10, FOS, PLG, and Ren, and the downregulated genes were MYC, TOP2A, FGA, CCNA2, and BUB1 (see Supplementary Tables S5, S6, and S11). Combining the data from the GO annotations and KEGG pathway enrichment analysis, the key differential genes for the occurrence and development of CKD may be ALB, MYC, IL10, PLG, REN, and FGA. The expression levels of the ALB, IL10, PLG, and REN genes in the CKD population was found to be significantly lower than that in the control group while the expression of MYC and FGA in the CKD population was significantly higher than in the control group.

## Discussion

Hyperuricemia is a systemic metabolic disease caused by disordered purine metabolism. Purines have important physiological functions in the human body, the most important of which is undoubtedly their incorporation with nucleic acids, thus forming part the genetic material in the body (Wang et al., 2022). Purines can exist as components of high-energy compounds such as adenosine triphosphate (ATP), which provide energy for different cellular activities (Wynne and Affourtit, 2022). In addition, purine bases can form part of cyclic adenylic acid (cAMP), cyclic guanylic acid (CGMP), and other second messenger substances that play key roles in the regulation of signal transduction (Illes et al., 2021). Finally, purines may also be components of some coenzymes that play key roles in the metabolism of sugar, fat, protein, and other important substances in the body (Patinha et al., 2020). Nucleic acids are abundant in the human body and do not require additional supplementation. Therefore, nucleic acids are not essential nutrients.

The kidney is the most important organ for excreting uric acid and excretes nearly two-thirds of the total uric acid produced. The remaining one-third is then excreted by the small intestine (Suijk et al., 2022). With the progression of CKD, the uric acid excreted by the digestive tract is greatly increased to maintain normal levels of uric acid in the blood. In addition, the synthesis of uric acid is correspondingly reduced by feedback inhibition (Radulescu et al., 2021; Ramos and Goldfarb, 2022). This study found that the intensities of the peak at 1,596 cm^−1,^ indicative of purines, were significantly lower in patients with stage 1 and stage 2 CKD combined with HUA than in the controls (see Figure 4A and Supplementary Tables S1, S2). Similarly, uric acid in urine samples is represented by the peak at 640 cm^−1^, which was observed to be significantly lower in the CKD groups in comparison with the control. These results were consistent with the trend of decreasing uric acid excretion in the urine of CKD patients with HUA, although no significant differences were observed between the three stages of the disease. This result suggests that uric acid is the end product of purine metabolism in human body and is mainly discharged through urine. HUA patients have a reduced ability to eliminate uric acid in their urine, probably due to higher levels of blood uric acid. At the same time, purine metabolism in patients with HUA is known to be abnormal, and this process may occur prior to obvious damage to renal function. In this context, analyzing the 1,596 cm^−1^ peak in urine may provide a reference for when to start drug treatment in patients with HUA.

The etiology of CKD is mainly secondary to diabetes and hypertensions in western countries. In contrast, in China, primary glomerulonephritis is still dominated by the highly common IgA nephropathy. The latter may be accompanied by diabetic nephropathy, hypertensive nephropathy, lupus nephritis, obstructive nephropathy, and polycystic kidney disease. During the early stages of CKD, the kidneys are able to compensate efficiently, allowing them to maintain the clearance of toxins and metabolites, body fluids, and solutes, thereby ensuring the stability of the internal environment. Due to the nearly normal functioning at this point, clinical symptoms are not obvious. However, when the GFR decreases progressively and the compensatory functions of residual nephrons can no longer meet the minimum requirements of the body, clinical symptoms will appear.

In this study, the internationally recognized eGFR standard was used to stage the patients with HUA. The level of blood uric acid in the control group was significantly lower than that of the test group, with no significant differences in the levels in patients in different CKD stages. It has been observed that routine indicators used for the clinical evaluation of renal function (blood urea, blood creatinine) and urine clinical data (urine microalbumin creatinine ratio, urine creatinine, urine microalbumin, and urine pH) were unable to distinguish between CKD patients. Kidneys have a strong reserve compensation capacity, and CKD is a slow and gradual process. Although the etiology is different, with increased age, the anatomical features and physiological metabolism of the kidneys undergo degenerative changes to varying degrees. Consequently, the GFR values of elderly patients tend to show a progressive downward trend. Subjects with primary renal diseases were not included in this study, while those who had undergone physical examination in the physical examination center were randomly selected for recruitment. At the same time, patients with basic renal disease were excluded. Therefore, the age of the patients with stage HUA combined with stage 3 CKD was higher, with a median of 80.5 years. Similarly, the median age of patients with HUA and stage 2 CKD was 76.5 years, while for the control, the median age was 90.50 years. There were no significant statistical differences between the three groups. The median age of patients with HUA combined with stage 1 CKD was 45 years old, which was significantly younger than the other three groups.

Previous studies on HUA have shown (Ortiz et al., 2022), that the prevalence of HUA in men was higher compared with women, and this is consistent with the inclusion of more male than female patients in this study. These observations could be attributed to the presence of obvious sex differences in HUA, with the blood uric acid level also being significantly higher in men than in women of the same age group. At the age of puberty, women have lower blood uric acid values due to estrogen which promotes the renal clearance of uric acid. The age of onset is also significantly later than that of men as, after menopause, the level of estrogen decreases significantly, reducing the uric acid clearance rate by the kidney, with corresponding increases in the blood levels of uric acid. Therefore, men are more likely to suffer from hyperuricemia than women. However, while this difference is obvious before menopause, it gradually diminishes after menopause. Therefore, women often suffer from hyperuricemia after menopause, which is related to the difference in sex hormone levels between men and women.

This study suggests that the pH of urine from non-HUA patients was significantly higher than that in the HUA group, with metabolic acidosis associated with the entire CKD process. More specifically, the pH of urine from stage 3 CKD patients with HUA was significantly lower than that of stage 1 and 2 CKD patients or even that of the control group. Although uric acid is more soluble in urine than in blood, the urine pH nevertheless influences the conversion rate of uric acid to urate as well as the solubility of urate in urine. For instance, in an alkaline environment, uric acid will be converted into urate because of its high solubility, thus promoting renal excretion and alleviating potential damage to the kidneys (Ramos and Goldfarb, 2022). In the Raman spectrum of urine, 640 cm^−1^ represents the peak position of uric acid, and the significantly lower levels in patients with CKD and HUA suggest that uric acid excretion is abnormal in patients with HUA. The determination of uric acid in the urine by Raman spectroscopy simplifies the determination process of uric acid and provides the possibility for the rapid and simple detection of uric acid.

In the Raman spectrum of urine, 1,608 cm^−1^ represents the peak position of urea. Urea levels in the urine of patients with stage 1 and stage 2 CKD are lower compared with the control group, while the urea level in stage 3 CKD patients did not differ significantly from that of the control group, which is itself consistent with the levels present in the peripheral blood among the groups. Regarding creatinine, the peak occurs at 1,706 cm^−1^ with the levels being significantly lower for all CKD subgroups in comparison with the control.

Hyperuricemia patients often present with complications resulting from dyslipidemia, especially from triglyceride (TG) abnormalities. Even in healthy people, it has been found that the level of blood uric acid is positively correlated with the triglyceride and cholesterol levels. Hence, increases in the levels of TG, TC, and LDL-C are seen as an independent risk factor for hyperuricemia. There is an apolipoprotein E2 allele in patients with hypertriglyceridemia and hyperuricemia, which reduces uric acid excretion by the kidneys, and the increased lipoprotein may lead to the reduction of blood uric acid clearance. It is speculated that TG may be a common factor that influences metabolism in hyperuricemia, and as such, controlling blood lipid metabolism disorders can effectively improve the process of hyperuricemia (Sultani et al., 2020). High triglyceride levels are a marker of impaired cellular energy metabolism. Since damaged cells have a poor ability to burn sugar, the body must maintain high TG levels in the blood as food for cells that cannot burn sugar. In this study, there were no significant differences in the levels of cholesterol, triglycerides, and glycosylated hemoglobin in the peripheral blood of the control group and the CKD subgroups (see Supplementary Tables S4, S5). However, through Raman spectroscopic analysis of the urine, it was noted that the urine of stage 1 CKD patients with HUA showed lower intensities of β-hydroxybutyric acid than the urine from patients with stage 2 and 3 CKD, with the difference being statistically significant (*p* < 0.01, see Supplementary Table S2). Similarly, when compared to the control, the urine of stage 1 CKD patients with HUA still contained significantly lower β-hydroxybutyric acid levels (*p* = 0.000, see Supplementary Table S2). Previous studies have shown that CKD patients have disordered energy metabolism (Liu et al., 2022), with low levels of β-hydroxybutyric acid (Galindo et al., 2021). Under physiological conditions, renal proximal tubules are mainly powered by fatty acid oxidation (Forbes and Thorburn, 2018). Patients with CKD have insufficient fatty acid oxidation due to kidney damage, and β-hydroxybutyric acid is a metabolic product of fatty acid oxidative decomposition. We speculate that the decrease in its content is related to the presence of dyslipidemia in CKD patients. β-Hydroxybutyric acid is the main component of ketone bodies, and the representative peak of urine is 1,643 cm^−1^, which is produced by ketogenic diets. β-Hydroxybutyric acid can not only be used as an energy supply fuel, but also can generate ATP through metabolism, and can also generate cellular signals (Strassheim et al., 2021). This cellular signal may help reduce oxidative stress in animals, which is one of the ways to delay aging (Park and Kim, 2020; Habieb et al., 2021). Insufficient oxidation of fatty acids in CKD patients causes lipid accumulation, which in turn leads to lipotoxicity. The latter is mainly characterized by mitochondrial dysfunction, increased production of reactive oxygen species (ROS), and decreased production of cellular ATP (Jao et al., 2019; Li M. et al., 2020; Thongnak et al., 2020). Ketone bodies can also competitively inhibit the ability of renal proximal convoluted tubules to secrete uric acid, thus resulting in an abnormal energy metabolism pathway that impacts uric acid levels. In this context, it was speculated that the renal cells can reduce ATP production as a result of lipotoxicity. However, β-hydroxybutyric acid compensates for increased production of ATP *in vivo* by counteracting oxidative stress and promote ATP consumption, thus explaining why β-hydroxybutyric acid (1,643 cm^−1^) in the CKD group was lower than that of the control group. In stage 1, CKD patients with HUA without obvious renal function impairment, have abnormal energy metabolism. At this time the β-hydroxybutyric acid concentrations are high, and the presence HUA in this period indicates that the body is protecting itself. However, CKD progression disrupts the internal microenvironment, reducing the protective and regulatory effects of HUA and thus inducing damage to the kidneys.

As research on human genomics has progressed, an increasing number of transporters or proteins regulating the function of uric acid transporters have been discovered. When the kidney is damaged, even if the glomerular filtration function is not affected, the damage of renal tubulointerstitium can also lead to changes in uric acid excretion fraction and ultimately affect the level of blood uric acid. The key genes for the occurrence and development of CKD were investigated through bioinformatics analysis before analysis of the GO functions and KEGG pathways associated with the DEGs. The principal DEGs were found to be *ALB, MYC, IL10, PLG, REN,* and *FGA*. Of these, *ALB* (albumin) is a protein synthesized by mammalian liver cells. As the most important protein in human plasma, it has important physiological functions such as the maintenance and stabilization of plasma osmotic pressure and participating in material transport. *ALB* gene expression is tissue-specific, that is, albumin can only be synthesized by hepatocytes, while the *ALB* gene in other tissues is not expressed. Albumin may be the main target of oxidative stress, and the anti-inflammatory and nephroprotective effects of dexamethasone are achieved by increasing two enhancers at the transcription start site of the *ALB* gene (Gong et al., 2020). IL-10 (interleukin-10) is a cytokine with anti-inflammatory properties. The dysregulation of IL-10 is related to a variety of kidney diseases. ReducedIL-10 expression can aggravate inflammatory activity, damage the metabolism of macrophages, and reduce the clearance of defective macrophages as well as the phagocytic capacity of macrophages (Ip et al., 2017). The plasminogen gene (*PLG*) encodes plasminogen, an enzyme secreted by the liver, which is converted into plasmin by a variety of enzymes when combined with blood clots. PLG plays an important role in tissue remodeling during growth and development, inflammation, body injury, and carcinogenesis. It can help degrade the extracellular matrix and matrix metalloproteinases (Rider et al., 2022). The main expression site of the *REN* renin gene is the paraglomerular cells of the kidney, which constitutes the renin angiotensin aldosterone system (RAAS) (Dilliott et al., 2020). As a pressor regulation system *in vivo*, angiotensin II binds to angiotensin II type 1 receptor (AGTR1) in blood vessels, adrenal glands, heart, central nervous system, and other tissues (Donlon and Morris, 2019), and plays a role in vasoconstriction and aldosterone secretion. It also plays a role in inflammation and sodium retention (Dilliott et al., 2020). *REN* gene mutations can cause abnormal protein accumulation in cells, leading to faulty renin production and apoptosis (Petrijan and Menih, 2019). In CKD patients, the expression of the above-mentioned *ALB, IL10, PLG*, and *REN* genes was significantly reduced compared with the control group. Based on the gene functional analysis, it was speculated that the occurrence and development of CKD could be related to oxidative stress, inflammatory factors, thrombosis, vascular expansion, and exudation (see Supplementary Figure S3). *MYC* is a proto-oncogene and a key regulator of cell proliferation that is involved in the repair of kidney injuries by regulating cell proliferation. Fibrinogen, encoded by the *FGA* gene, is composed of three chains. As one of the main components of blood clots, fibrinogen plays an important role in hemostasis. Mutations in *FGA* are related to renal amyloidosis (Li F. et al., 2020). In CKD patients, the upregulation of *MYC* and *FGA* is one of the reasons for the aggravation of renal damage. According to the gene functional analysis, we speculate that the occurrence and development of CKD may be related to self-repair defects and amyloidosis (see Supplementary Figure S3).

Based on the results of the bioinformatics analyses, CKD progression is closely related to oxidative stress, inflammatory reactions, and thrombosis. Since purines are involved in the formation of energy-containing substances and signal transduction, they can participate in metabolism. Therefore, they are likely to play a key role in the process of kidney diseases. In this study, OPLS-DA was first used to establish a model, and the possible differences peak positions were found through multi-dimensional calculation, before further verification using single-dimensional statistics. It was found that the 828-cm^−1^ peak position representing glutathione/tryptophan, the 1,556-cm^−1^ peak position representing tryptophan, the 1,585-cm^−1^ peak representing C-C bonds, and the 1,587-cm^−1^ peak representing tyrosine played important roles in the identification of CKD samples. Glutathione is the most important antioxidant in human body. After oxidation of the carbon-carbon double bond, the double bond breaks. Tryptophan and tyrosine can then act as glycogenic and ketogenic amino acids and participate in the body’s energy metabolism. Although there were no significant differences between the CKD subtypes with HUA, the peak positions related to antioxidation and energy metabolism in the urine of stage 3 CKD patients with HUA showed an increase, suggesting that there may be poor energy conversion and weakened antioxidant capacity in stage 3 CKD patients (see Supplementary Tables S1–S3).

HUA is a risk factor for CKD, and it is thus very important to control blood uric acid to delay the progression of kidney disease. There are a large number of asymptomatic HUA patients in the clinic. The cause of HUA is related to purine metabolism. Purine plays an important role in maintaining human physiological function. Excessive uric acid lowering treatment will inevitably lead to abnormal purine metabolism in the body. Therefore, there is no unified view on when to start the intervention treatment of hyperuricemia. In Japan, for patients with stage 3 CKD with glomerular filtration rate (eGFR) < 60 mL min^−1^·1.73 m^−2^, the treatment of reducing blood uric acid is considered to be beneficial for the control of kidney disease, and it can delay the progression of CKD and prevent cardiovascular events by reducing uric acid level (Nakaya et al., 2011). Using urine sample collection, the analysis of Raman spectra, and multivariate analysis to establishe an identification model, this study has formed a complete set of identification research ideas for CKD. In patients with stage 3 CKD combined with HUA, the changes in these peaks were not prominent. The causes of renal injury in patients with stage 3 CKD are complex, and it is very difficult to determine renal function only through urine. We believe that for patients with stage 1 and 2 CKD combined with HUA, the use of the spectral data of β-hydroxybutyric acid (1,643 cm^−1^ peak), purine (1,596 cm^−1^ peak), glutathione/tryptophan (828 cm^−1^ peak), tryptophan (1,556 cm^−1^ peak), carbon-carbon bonds (158 cm^−1^ peak), and tyrosine (1,587 cm^−1^ peak) is simple and easy to use. It can assess the presence of abnormal energy metabolism in patients with HUA and dynamically determine the degree of renal function damage in patients with HUA, thus providing a reference for finding a reasonable time for uric acid intervention. The limitation of this study is that the sample size was small, and a large number of carefully designed prospective studies are still needed to confirm the time sequence and causal relationship between hyperuricemia and CKD to lay a foundation for exploring the changes in the molecular environment of the urine of CKD patients with HUA. Interventions to modulate the internal environment and energy metabolic signaling levels may to delay renal injury in patients with HUA.

The innovation of this study lies in that, firstly, compared with traditional methods, Raman spectroscopy technology is simple, fast, and accurate for the capture of biomolecular “fingerprints” in the urine of patients with HUA; a single integration time for a spectrum is only 250 s, no expensive reagents and substrates are needed, and the cost is low. Secondly, the urine sample is easy to obtain and does not harm the patient and can thus be used as a supplement for the clinical detection of renal system diseases. Thirdly, the combination of Raman spectroscopic data and bioinformatics analysis explored the genetic basis relating to oxidative stress, inflammatory reactions, and thrombosis associated with CKD progression. Fourthly, comparing the OPLS-DA model analysis established by massive biochemical data, establishing the identification model of CKD population combined with HUA will help improve the timeliness of renal system disease screening and reduce the economic burden of patients. Fifthly, the results of the combination of Raman spectroscopic technology and clinical data can be developed into home portable instruments to monitor specific indicators as an auxiliary tool for kidney disease inspection, which is of great significance for the early assessment of patient prognosis and the reduction of the economic burden of patients with end-stage kidney disease.

Compared with traditional technologies, Raman spectroscopy has the advantages of being non-invasive, having easy access to specimens, and providing real-time reflections of body conditions. It is convenient for repeated sampling to monitor the dynamic development of diseases, and Raman data provide a large amount of information that can quantitatively reflect the concentrations of substances, which is of great clinical significance for the early screening stage of diseases. In the past, Raman spectroscopy has been used to study blood samples, but rarely urine samples. At present, the analysis of urine components of asymptomatic patients with HUA and CKD is still undocumented. At present, few studies have combined the use of Raman spectroscopy with clinical data, nor of Raman spectroscopy with bioinformatics analysis. This study is thus innovative in combining Raman spectroscopy, clinical data, and bioinformatics analysis to more comprehensively analyze the metabolic characteristics and pathogenesis of patients with HUA combined with CKD, for a comprehensive analysis of the characteristics of these patients, allowing an easy means of early detection, intervention, and treatment, thus reducing the economic burden on patients and society.

To sum up, it is very important to start treatment before HUA patients have developed gout symptoms and before complications arise due to CKD, especially as HUA patients may have abnormal energy metabolism. Raman spectroscopy was applied to interpret the heterogeneity of the urine environment of HUA patients. At the same time, combined with an OPLS-DA model, clinical and biochemical data of HUA patients was mined to explore the close relationships between the progression of CKD and oxidative stress, inflammatory reactions, and thrombus as well as to explore the peak intensities of β-hydroxybutyric acid, purine, glutathione, tryptophan, and tyrosine can help to determine the degree of renal function damage in CKD patients with HUA and provide a reference for finding a reasonable time for uric acid intervention. The collection of urine samples is simple and easy. Monitoring the changes of urine microenvironment of CKD patients with HUA by Raman spectroscopy can provide help for early detection of changes in human internal environment, provide a new idea for exploring a low-cost and rapid assessment of renal function of patients with HUA and CKD, and also improve the timeliness level of CKD disease screening, reduce the economic burden of patients, It provides a useful technical supplement for the accurate diagnosis of chronic kidney disease. Precise intervention of abnormal signal levels of internal environment and energy metabolism may be a potential way to delay renal injury in patients with HUA.

## Data Availability

Publicly available datasets were analyzed in this study. This data can be found here: https://www.ncbi.nlm.nih.gov/geo/ - GSE66494.
