# Supplementary material for: Rapid identification of early renal damage in asymptomatic hyperuricemia patients based on urine Raman spectroscopy and bioinformatics analysis
Source: Front Chem. 2023 Jan 25;11:1045697. doi: 10.3389/fchem.2023.1045697 (PMC9905717; doi:10.3389/fchem.2023.1045697)
Supplement: Supplementary file 1 [file DataSheet1.pdf]

Supplementary Material

Table S1. Results of screening for statistically significant potential biomarker peaks for control and CKD groups

Peaks (/cm^{-1})	control group	stage 1 CKD	stage 2 CKD	stage 3 CKD	*P* value
640	148.66(128.05-171.51)	110.54(48.17-120.39)	91.26(52.14-137.84)	94.38(43.04-134.86)	0.009
642	147.88(129.81-164.17)	108.35(50.76-121.73)	95.87(52.92-140.81)	94.96(46.12-133.77)	0.014
828	336.57±41.95	239.39±30.68	213.42±45.18	201.09±48.15	0.115
1556	44.21±4.28	23.60±3.36	18.89±3.54	24.57±8.21	0.001
1585	80.87(57.16-102.51)	35.41(28.73-56.57)	30.63(17.86-48.81)	64.84(16.36-85.59)	0.011
1587	83.17(60.42-105.42)	37.12(23.15-53.67)	36.63(17.86-53.50)	65.86(17.58-93.67)	0.009
1596	66.10(37.84-98.36)	16.97(9.12-55.04)	17.31(8.52-34.65)	75.34(14.73-102.35)	0.012
1603	112.66(76.59-129.34)	29.20(21.66-71.08)	37.17(27.18-64.11)	101.49(27.19-144.69)	0.010
1615	86.64±6.75	56.21±5.45	47.25±6.18	84.49±21.70	0.005
1608	92.89(72.63-112.48)	32.12(20.60-60.28)	37.74(23.93-54.65)	86.13(23.07-113.02)	0.004
1706	58.25(53.86-76.84)	37.66(20.98-51.04)	31.98(14.13-42.53)	38.83(9.66-60.61)	0.002
1643	85.31±3.29	63.06±2.29	73.11±1.25	78.62±4.22	0.000

Note: Data conforming to normal distribution are expressed as mean ± standard deviation. Data not conforming to the normal distribution are expressed using M(Q1-Q3).

Table S2. Results of screening for statistically significant potential biomarker peaks in subjects at different CKD stages

groups	peaks (/cm^{-1})	materials	*P* value						references
			stage 1 CKD and stage 2 CKD	stage 1 CKD and stage 3 CKD	stage 2 CKD and stage 3 CKD	stage 1 CKD and control group	stage 2 CKD and control group	stage 3 CKD and control group	
Uric acid	640	Uric acid	0.698	0.511	0.759	0.007	0.005	0.006	1
Proteins and amino acides	642	Tyrosine	0.775	0.612	0.804	0.008	0.007	0.010	2
	828	Glutathione / Tryptophan	0.623	0.540	0.853	0.072	0.039	0.048	1,2
	1556	Tryptophan	0.413	0.886	0.435	0.001	0.000	0.010	2
	1585	C=C Olefifinic Stretch	0.368	0.461	0.161	0.011	0.002	0.183	3
	1587	Proteins / Tyrosine	0.517	0.357	0.169	0.005	0.002	0.180	2
	1596	Alanine/Serine	0.859	0.073	0.069	0.007	0.008	0.643	1
	1603	Tryptophan/ Phenylalanine	0.754	0.064	0.138	0.003	0.014	0.514	2
	1615	Tyrosine / Tryptophan	0.871	0.830	0.627	0.014	0.003	1.000	3
Urea and creatinine	1608	Urea	0.838	0.086	0.149	0.001	0.005	0.327	4,5
	1706	creatinine	0.398	0.931	0.454	0.003	0.000	0.021	1
Ketone bodies	828	β-Hydroxybutyrate	0.623	0.540	0.853	0.072	0.039	0.048	1
	1643	β-Hydroxybutyrate	0.007	0.001	0.224	0.000	0.003	0.141	5

Table S3. Comparison of clinical data from subjects assigned to different CKD stages, combined with hyperuricemia and control group

	control group	stage 1 CKD	stage 2 CKD	stage 3 CKD	*P* value
Cases	10	15	10	6	
Age	90.50(88.00-92.25)	45.00(39.00-54.00)	76.50(60.75-80.25)	80.50(69.00-93.25)	0.000
Gender(female/male)	8/2	15/0	9/1	3/3	0.030
BMI(kg/m2)	23.4(22.04-24.83)	24.62(22.72-26.18)	27.75(23.63-28.87)	24.31(22.78-25.89)	0.073
Uric acid (umol/L)	259.00±88.64	486.33±50.31	486.00±45.49	484.17±67.40	0.000
eGFR[ml/(min ·1.73m^2)]	87.73±18.78	108.25±12.08	81.31±6.50	56.47±3.14	0.000
Urea(mmol/L)	6.40(4.70-10.08)	4.40(3.90-4.80)	4.70(4.20-6.05)	6.95(6.55-8.30)	0.003
Creatinine (umol/L)	54.50(48.25-67.75)	72.00(69.00-75.00)	80.50(68.00-85.50)	95.00(83.50-107.50)	0.001
ACR	42.95(14.54-212.78)	2.58(2.19-3.83)	5.07(2.82-11.74)	27.08(8.61-60.85)	0.004
UCR (umol/L)	4403.80±2240.76	9418.13±4682.85	7546.70±5502.53	6332.23±2091.00	0.043
m-ALB((mg/L)	16.83(8.26-34.00)	3.6(1.80-4.50)	4.6(1.70-13.48)	16.85(6.88-38.26)	0.010
Urine pH	7.25(6.00-8.13)	6.50(6.00-6.50)	6.25(6.00-6.63)	6.00(5.50-6.13)	0.025
SG	1.015(1.013-1.018)	1.015(1.010-1.020)	1.013(1.010-1.016)	1.013(1.010-1.017)	0.766
TP(g/L)	65.30±8.11	73.40±4.42	73.00±4.32	67.66±6.92	0.006
ALB(g/L)	32.70±7.04	45.27±1.87	44.80±2.15	40.17±6.65	0.000
GLO(g/L)	32.60±5.91	27.20±5.87	28.20±3.49	27.50±1.05	0.062
ALT(U/L)	18.50(10.50-27.50)	24.00(21.00-30.00)	22.00(17.75-28.00)	21.00(15.75-26.75)	0.331
TBIL(umol/L)	10.06±4.85	15.71±4.05	15.44±4.04	12.40±6.57	0.023
DBIL (umol/L)	3.82±2.63	4.02±1.52	4.45±1.60	3.30±1.81	0.695
GLU(mmol/L)	5.10(4.80-5.93)	4.80(4.50-5.30)	5.60(5.28-6.13)	6.05(4.63-6.98)	0.019
HbA1c (%)	5.89±0.63	5.81±0.33	6.17±0.73	5.97±0.98	0.561
TC(mmol/L)	3.96±1.10	5.01±0.64	5.12±1.30	4.74±0.96	0.046
TG (mmol/L)	1.43(1.03-1.99)	1.92(1.27-2.84)	1.60(1.34-2.42)	1.86(1.12-2.64)	0.612
HDL-C (mmol/L)	1.05±0.24	1.14±0.13	1.14±0.15	1.21±0.42	0.565
LDL-C (mmol/L)	2.13±0.55	3.07±0.56	3.22±1.03	2.63±0.84	0.009
AFP (ng/mL)	2.06(1.04-2.57)	2.91(1.86-4.10)	2.57(2.17-2.71)	1.72(1.48-2.15)	0.017
CEA(ng/mL)	3.95±2.46	1.28±0.57	2.64±1.11	3.80±2.11	0.001
RBC (*10^{12}/L)	3.76(2.72-4.04)	5.09(4.81-5.34)	5.08(4.91-5.16)	4.42(3.52-5.06)	0.001
HGB (g/L)	109.60±26.98	153.13±8.32	149.50±12.25	133.33±21.43	0.000
HCT (%)	32.70±7.71	45.49±2.94	45.12±2.51	38.62±7.34	0.000
MCV (fl)	93.40(91.30-98.98)	90.30(86.70-96.60)	90.85(87.13-94.75)	90.75(88.60-95.50)	0.385

Table S3. Comparison of clinical data from subjects assigned to different CKD stages, combined with hyperuricemia and control group (continued)

	control group	stage 1 CKD	stage 2 CKD	stage 3 CKD	*P* value
MCHC (g/L)	321.50(315.25-341.75)	333.00(325.00-340.00)	331.50(325.75-339.25)	332.50(321.25-341.75)	0.071
MCH(pg)	30.55(29.15-31.28)	30.00(29.20-31.60)	30.15(28.65-32.38)	30.55(29.70-39.18)	0.940
RBC-CV (%)	14.20(12.95-16.13)	12.40(12.20-12.70)	13.05(12.48-14.28)	12.25(12.00-13.60)	0.009
RBC-SD (fl)	48.65(43.25-55.75)	41.10(39.10-41.90)	42.25(40.53-45.20)	41.15(39.48-45.73)	0.003
WBC (*10^9/L)	6.92(6.15-8.49)	6.16(4.83-6.95)	6.88(4.69-7.47)	6.15(5.67-6.58)	0.244
NEU (%)	62.96±9.82	52.57±7.85	60.20±8.34	58.37±6.39	0.024
NEU# (*10^9/L)	4.74±1.90	3.16±0.84	3.79±1.18	3.52±0.41	0.027
LYM (%)	22.83±8.66	36.45±6.64	29.93±9.40	29.08±4.43	0.001
LYM# (*10^9/L)	1.65±0.59	2.16±0.53	1.82±0.55	1.87±0.25	0.113
MON (%)	7.80(7.68-8.93)	7.10(6.40-8.60)	7.70(6.90-8.50)	9.45(8.55-10.50)	0.022
MON#(*10^9/L)	0.56(0.48-0.70)	0.44(0.33-0.50)	0.52(0.36-0.60)	0.61(0.53-0.72)	0.022
EOS(%)	2.65(1.73-4.63)	2.30(1.70-3.60)	1.90(1.25-2.53)	4.10(2.28-4.40)	0.110
EOS# (*10^9/L)	0.22(0.14-0.36)	0.15(0.08-0.21)	0.12(0.08-0.14)	0.25(0.13-0.26)	0.033
BAS(%)	0.35(0.20-0.83)	0.40(0.40-0.70)	0.35(0.30-0.53)	0.65(0.53-0.83)	0.263
BAS#(*10^9/L)	0.04(0.02-0.05)	0.03(0.02-0.04)	0.02(0.02-0.03)	0.04(0.02-0.04)	0.328
PLT(*10^9/L)	200.60±11.01	229.87±65.57	175.80±53.92	210.50±57.09	0.137
PDW(fl)	12.83±2.24	11.50±1.22	12.37±1.53	12.08±3.37	0.412
FL(fl)	10.75±0.85	10.01±0.70	10.59±0.76	10.53±1.46	0.192
PCT(%)	0.22±0.04	0.23±0.06	0.19±0.04	0.22±0.06	0.346
P-LCR(%)	30.64±7.16	25.57±5.42	29.67±6.31	29.02±12.28	0.335

Note: Data conforming to normal distribution are expressed as mean ± standard deviation. Data not conforming to the normal distribution are expressed using M(Q1-Q3).

Table S4. Multiple comparison of clinical data between groups at different CKD stages combined with hyperuricemia and a control group

	P value					
	stage 1 CKD and stage 2 CKD	stage 1 CKD and stage 3 CKD	stage 2 CKD and stage 3 CKD	stage 1 CKD and control group	stage 2 CKD and control group	stage 3 CKD and control group
Age	0.007	0.001	0.388	0.000	0.074	0.495
BMI(kg/m2)	-	-	-	-	-	-
Uric acid (umol/L)	0.990	0.944	0.955	0.000	0.000	0.000
eGFR[ml/(min ·1.73m^2)]	0.000	0.000	0.000	0.050	0.908	0.003
Urea (mmol/L)	0.332	0.002	0.030	0.005	0.096	0.466
Creatinine (umol/L)	0.190	0.003	0.082	0.161	0.013	0.000
ACR	0.191	0.004	0.099	0.002	0.117	0.771
UCR(umol/L)	0.948	0.270	0.991	0.010	0.537	0.501
m-ALB(mg/L)	0.546	0.008	0.046	0.008	0.063	0.702
Urine pH	0.806	0.052	0.105	0.124	0.104	0.002
SG	-	-	-	-	-	-
TP(g/L)	0.868	0.050	0.087	0.002	0.006	0.440
ALB(g/L)	0.995	0.535	0.628	0.002	0.002	0.294
GLO(g/L)	0.625	0.901	0.786	0.011	0.055	0.054
ALT(U/L)	-	-	-	-	-	-
TBIL(umol/L)	0.889	0.151	0.215	0.005	0.014	0.337
DBIL(umol/L)	0.586	0.436	0.249	0.795	0.464	0.600
GLU(mmol/L)	0.005	0.027	0.863	0.068	0.359	0.535
HbA1c(%)	0.660	0.999	0.999	0.999	0.936	1.000
TC(mmol/L)	1.000	0.990	0.987	0.096	0.245	0.659
TG(mmol/L)	-	-	-	-	-	-
HDL-C(mmol/L)	1.000	0.999	0.999	0.896	0.922	0.963
LDL-C(mmol/L)	0.999	0.860	0.805	0.003	0.066	0.806
AFP(ng/mL)	0.638	0.007	0.032	0.026	0.108	0.449
CEA(ng/mL)	0.023	0.180	0.830	0.045	0.627	1.000
RBC(*10^{12}/L)	0.578	0.033	0.121	0.000	0.003	0.319
HGB(g/L)	0.964	0.367	0.581	0.003	0.006	0.372
HCT(%)	1.000	0.356	0.406	0.003	0.003	0.633

Table S4. Multiple comparison of clinical data between groups at different CKD stages combined with hyperuricemia and a control group (continued)

	P value					
	stage 1 CKD and stage 2 CKD	stage 1 CKD and stage 3 CKD	stage 2 CKD and stage 3 CKD	stage 1 CKD and control group	stage 2 CKD and control group	stage 3 CKD and control group
MCV(fl)	-	-	-	-	-	-
MCHC(g/L)	-	-	-	-	-	-
MCH(pg)	-	-	-	-	-	-
RBC-CV(%)	0.069	0.745	0.082	0.003	0.313	0.009
RBC-SD(fl)	0.154	0.628	0.501	0.000	0.042	0.015
WBC($*10^9$/L)	-	-	-	-	-	-
NEU(%)	0.031	0.158	0.672	0.004	0.463	0.292
NEU#($*10^9$/L)	0.659	0.752	0.987	0.168	0.735	0.382
LYM(%)	0.045	0.055	0.832	0.000	0.046	0.124
LYM#($*10^9$/L)	0.112	0.247	0.853	0.021	0.479	0.425
MON(%)	0.630	0.003	0.017	0.125	0.336	0.121
MON#($*10^9$/L)	0.435	0.011	0.078	0.016	0.135	0.639
EOS(%)	-	-	-	-	-	-
EOS#($*10^9$/L)	0.144	0.275	0.030	0.134	0.007	0.869
BAS(%)	-	-	-	-	-	-
BAS#($*10^9$/L)	-	-	-	-	-	-
PLT($*10^9$/L)	0.022	0.474	0.233	0.204	0.323	0.731
PDW(fl)	0.623	0.999	1.000	0.505	0.996	0.998
FL(fl)	0.120	0.233	0.902	0.049	0.689	0.639
PCT(%)	0.075	0.665	0.304	0.560	0.262	0.955
P-LCR(%)	0.505	0.990	1.000	0.376	1.000	1.000

Note: The data of BMI, SG, ALT, TG, MCV, MCHC, MCH, WBC, EOS, BAS, and BAS# did not conform to normal distribution and the overall nonparametric test did not detect significant differences between the samples, so no multiple comparisons were performed.

Table S5. Identification of up-regulated DEGs in gene chip GSE66494

Gene symbol (up-regulated DEGs)
CELA3B, ITLN1, S100P, REG1B, DHRS2, DHRS2, LMO7DN, GSTT1, RPS4Y2, RPS4Y1, MKL2, CTRB1, REG3A, GSTT1, JAKMIP3, CSE1L-AS1, UPK1A, PFKFB3, CIDEA, OR4C46, FOXR1, THEG, REG1A, CPB1, DES, PDXDC2P, LOC100507537, CROCCP3, SAA1, DDX3Y, PNLIPRP2, PDXDC2P, MALAT1, SLC12A3, NPHS1, RAB24, PXN, RGS1, ACTG2, GUSBP1, CPA1, PRSS1, SLC12A3, RGMA, HSPA1A, SLC14A1, NPHS1, SLC12A3, HRASLS2, KIAA1161, REN, KLK1, CAPN12, REN, PNLIP, PRODH2, METTL3, NEAT1, SMG1P5, PLIN1, PDE7A, SOST, ATG9B, SMG1P5, PCOLCE2, LOC645321, MLXIPL, SLC4A9, PRSS2, CALCA, RGS11, MFAP5, PABPC1L, MEG3, GUSBP1, ARHGAP1, ASS1, LPIN1, PILRB, HBD, NPHS2, ALB, TAC3, MCF2L, TRPM6, SLC26A10, JADE2, MAGEC1, PCBP1-AS1, SPINK1, FOS, CIRBP, LOC101927948, HSPA1A, AMY1C, ZDHHC11, PAGE4, FAM95A, PILRA, PFKFB3, PLG, DPEP1, SAPCD1, GOLGA8A, NPFF, ACSM2A, HRASLS2, MDH1B, RXRA, EPO, IGLL1, CYP2D6, KRT13, KIFC2, PDZD3, LINC00473, ST18, CIRBP, HEMK1, CAPN12, CCNL2, TRIM50, SLC22A6, PITX1, CYP27B1, ADD1, LOC105370792, HOXB8, UPK3A, SORCS1, PCP4L1, CCDC71, TANC2, SERPINF2, HBB, FOXA1, AHCY, SPAG9, TAPT1-AS1, PI4KAP2, FLJ36840, CYP2E1, FAM95B1, LOC101928658, FMN2, CHP2, TNFAIP8L3, HBA2, MLXIPL, FTCD, PCK2, LINC00265, ATP1A4, MPP3, GPD1, CRYAA, HBA2, THRSP, EVL, RNU12, HEMK1, LINC00982, PABPC1L, GNG13, ACOT11, GAD1, CRYAA, SEC31B, PRKCZ, LOC155060, CCDC18-AS1, BMP3, TRPM3, POU5F1, SPTBN5, NR4A1, HBA1, LOC101928524, FLNB, KCNH6, SNX31, NUP210, GOLGA8A, ASMTL-AS1, TNNT2, LGALS12, AATK, TXLNGY, ANP32A-IT1, DNAH1, GGT7, EIF1AY, LOC100190986, SLC26A6, CHI3L1, SIMC1, P2RX1, EEF1D, PTGS2, SCUBE2, CCDC80, SLC22A7, ALOX12P2, GRIP2, KIAA0319L, SLFNL1, MRC2, IDO2, NRBP2, TDRD10, TBC1D3B, RBBP6, PLA2G1B, LOC100190986, ANKRD20A5P, LY6G5C, POU5F1, CASC2, FLCN, KCNQ1OT1, CLIC5, MIR503HG, PCDHB9, LINC00342, TNNT2, SENP3, POTEM, TSC22D3, ACSS1, UPB1, PTPRG, PDXK, RDH12, B4GALNT3, SNHG8, RASD2, HPD, TMEM259, NR4A3, DLST, CYR61, LOC105376790, GOLGA8A, AKAP8L, AZGP1, CLASRP, SEC24C, GOLGA8F, ZNF493, FAM65C, LINC00839, ANGPTL7, PLG, SNORA70, PROZ, SERPINA4, ALS2CL, AVIL, PTPRO, GOLGA6C, ARHGAP24, MUC5B, PRSS50, ADIPOQ, KRT25, MELTF, EPS15L1, ERVH-4, ULK3, NSUN5P2, USP6, MYO15B, ARHGEF25, PGGHG, LOC100190986, SCARA5, KIAA0895L, ITIH4, ACCS, LINC00265, COL11A2, RUSC1-AS1, PHF21B, HERC2P7, SUN2, MPP3, SPG7, AFM, GOLGA6A, MOGAT1, EME2, ALLC, REG3G, LINC00955, POU5F1P4, HNRNPDL, SORBS1, ERVH-3, ALS2CL, NOC2L, SLC26A6, GAS6, ITM2C, MOGS, LUC7L3, CSRP1, SORBS2, ANXA6, C9orf47, ETNK2, DOCK4, MME, SLC25A27, FBLN1, DDX56, RIN3, CELA2B, ZNF767P, SPAG8, LINC00839, GUSBP4, CYP11A1, EPOR, MTHFR, SLC7A10, FAM219B, CSAG4, SELENBP1, GUSBP3, STRA6, SAA2, SPOCK2, CCDC57, ZDHHC11, LINC01578, FGFR3, POU5F1, SEMA4C, CAMSAP1, USP30, RRN3P2, TNNT2, FAM13A-AS1, CRYGS, CCNL2, NGFR, USH1C, WT1-AS, LOC146880, TMEM139, CLIC5, SMA4, GOLGA8F, GLRA3, WDR27, SLC28A1, GPATCH2L, CELF6, ZNF175, BCAR1, LOC202025, KIAA0485, CALD1, MICAL3, SLC28A2, KRT5, ANKRD20A2, AGPAT4-IT1, PEAR1, GOLGA8T, ZNF207, CUZD1, POLR3E, GNRH1, CAPN8, DUSP13, COLQ, GBA2, ATAD3B, TMPRSS5, TAS2R19, LOC100133331, RGMB-AS1, SLC23A3, HDAC10, CKM, LOC100506557, PRKAG2, GON4L, KCNK3, PILRB, FAM118A, ZYG11A, SMG1P1, KCNH6, GOLGA6L10, EHD3, CLUHP3, GP2, WFIKKN2, GYG2, MED26, IL10, SHISA7, ZNF692, AGXT, FKBP10, GSDMB, CCDC17, DUSP2, B4GALT2, NEAT1, NDRG2, SFXN4, BAIAP2, ZNF493, ZFY, SHANK1, GCGR, ACSF2, AQP3, GUSBP3, BTNL9, ACY3, ADAMTSL4, TCF21, SPPL3, LOC101928291, STAG3L3,

GPR157, C2orf54, LINC00473

Table S6. Identification of down-regulated differentially expressed genes in gene chip GSE66494

Gene symbol (down-regulated DEGs)
PKD2L2, AKR1B10, SYCE3, GPNMB, NKAIN4, CD1A, TDRD12, BUB1, COL17A1, LOC100507388, SLC27A1, CCAT1, SMG8, BHLHE22, CABP7, SVOP, LOC101927588, FAM30A, UGT2B4, E2F2, TRIM6, FGA, NLN, DIAPH3, LRAT, CABYR, SLC38A4, IL27, KIF20A, SPAG1, AQP4, TRIM6, HOXC11, OSMR, MPZL2, AQP9, AGR3, SAMD10, IL36RN, FAM84A, CROT, DEFB104B, CNIH2, GBP6, VCAM1, GPRIN1, DLGAP5, PCDHB16, TRIM59, CXCR5, ZDHHC13, LBX2, SOX9, CTAG1A, CCL20, CLC, CENPF, TNFSF10, LIN28A, FGF16, QPCT, FAM135B, SECTM1, ALPPL2, CASC9, TP53TG3, NCAPG, SLC34A2, ZIC4, TAC1, FGF7P6, LAMC2, CXorf36, ARNTL2, WFDC2, MYC, MELK, TAC1, ANLN, BEAN1, CLECL1, DSCR8, SCARNA17, BID, CDKAL1, MAP9, C20orf85, XIRP2, POLR2J2, KCTD4, KIR2DS4, C15orf48, CCNB2, ITGB3, IKBIP, SYT16, KCNQ2, FGF7P6, WFDC2, DCTN1, ASB16, SPC25, TOP2A, QPCT, BEST3, LCAL1, CHI3L2, CXCL6, SPON2, GDF6, DNAH5, FOXC2, ESCO2, MUC13, MIR7-3HG, CDH22, KCNIP4, SPINK2, KRTAP17-1, IL4I1, DTL, KCNIP4, ADAMTSL5, LYZ, LOC100507412///RNA5-8S5, SOX4, MCTP1, MAB21L2, KIF25-AS1, ITGB6, SIAE, NKX6-2, HLA-DQA1, CYP4F11, NOL6, CEP55, OR5L2, TUBB3, CST6, PBK, MMP7, EDN2, FBLL1, LTF, FGB, C5orf58, LOC100996579, CYP4F11, XIST

Table S7. Top 5 data for biological process enrichment analysis of CKD DEGs

Category	Term	Count	%	*P* Value	Genes
Biological Process	cell wall disruption in other organism	4	0.738007	0.000051	REG3A, REG1B, REG1A, REG3G
Biological Process	acute-phase response	8	1.476014	0.000052	ITIH4, REG3A, EPO, SERPINF2, SAA1, SAA2, REG3G, ASS1
Biological Process	water transport	6	1.107011	0.000083	SLC14A1, PDZD3, AQP9, AQP4, UPK3A, AQP3
Biological Process	oxygen transport	5	0.922509	0.000342	MYC, HBB, HBA2, HBD, HBA1
Biological Process	proteolysis	23	4.243542	0.000856	CELA3A, PRSS1, CPA1, GGT7, CPB1, CELA2B, MMP7, TMPRSS5, MME, CTRB1, PROZ, PLG, SPG7, WFDC2, NLN, CAPN8, PRSS50, CAPN12, QPCT, DPEP1, REN, PRSS2, LTF

Table S8. Top 5 data for cellular component enrichment analysis of CKD DEGs

Category	Term	Count	%	*P* Value	Genes
Cellular Component	extracellular space	80	14.760147	0.00000019	PNLIPRP2, CXCL6, PRSS1, EPO, REG1B, WFIKKN2, REG1A, PRKAG2, HBB, SECTM1, LAMC2, IL27, SERPINA4, WFDC2, ACTG2, MCF2L, TNFSF10, MUC13, SOST, GUSBP3, PRSS2, IL10, FGB, FGA, AMN, CPA1, CELA2B, MMP7, EDN2, NPFF, RALGAPA2, AMY1C, ADIPOQ, CTRB1, SERPINF2, HBA2, HBA1, AZGP1, ANGPTL7, CHI3L2, DPEP1, CHI3L1, GAS6, PNLIP, LTF, COL17A1, CELA3A, REG3A, CALCA, CPB1, PLA2G1B, COL11A2, PROZ, AFM, ITLN1, FBLN1, PLG, REG3G, CD1A, SCUBE2, SELENBP1, IL36RN, TAC3, SIAE, TAC1, VCAM1, CKM, CCL20, SEMA4C, GP2, GDF6, MUC5B, LYZ, COLQ, BMP3, FGF16, ALB, GNRH1, REN, MELTF
Cellular Component	extracellular region	85	15.682656	0.00000046	PNLIPRP2, CXCL6, PRSS1, EPO, WFIKKN2, HBB, LAMC2, AQP4, IL27, SERPINA4, WFDC2, IL4I1, ADAMTSL5, ADAMTSL4, TNFSF10, SOST, CCN1, PRSS2, IL10, FGB, FGA, SPINK2, CELA2B, MMP7, EDN2, DIPK2B, NPFF, ADIPOQ, CTRB1, SERPINF2, HBA2, HBA1, AZGP1, CCDC80, ANGPTL7, CHI3L2, CHI3L1, GAS6, PNLIP, LTF, COL17A1, LRPAP1, REG3A, ITIH4, CALCA, PDXK, DNAH1, PCOLCE2, PLA2G1B, DNAH5, COL11A2, PROZ, AFM, ITLN1, FBLN1, PLG, REG3G, SCUBE2, NLN, SPOCK2, IL36RN, TAC3, TAC1, NGFR, DEFB104A, CCL20, GP2, GDF6, LYZ, EPOR, MFAP5, BMP3, FGF16, CABYR, AKR1B10, IGLL1, QPCT, ALB, SAA1, GNRH1, REN, MELTF, LY6G5C, FGFR3, HSPA1A
Cellular Component	extracellular exosome	84	15.498154	0.00000558	CLIC5, SPON2, ACY3, REG1B, ITGB3, PTPRO, REG1A, ARHGAP1, HBB, SECTM1, JADE2, SERPINA4, WFDC2, ACTG2, FTCD, PTPRG, UPK1A, CSRP1, TUBB3, KRT25, TNFSF10, ANXA6, GBP6, FGB, FGA, AMN, SPINK1, MMP7, MME, AMY1C, ACOT11, SERPINF2, MOGS, HBA2, KRT5, HBA1, UPB1, UPK3A, AZGP1, GPD1, DPEP1, CHI3L1, GAS6, HPD, LTF, ITIH4, SLC22A6, PDXK, AHCY, KLK1, PROZ, AFM, ITLN1, GSTT1, FBLN1, PLG, NDRG2, CST6, PRKCZ, SELENBP1, FLNB, SIAE, SPAG9, SLC12A3, VCAM1, KRT13, GP2, PILRA, MUC5B, BAIAP2, LYZ, ASS1, BMP3, DES, NPHS1, NPHS2, QPCT, ALB, SAA1, SAA2, MELTF, PABPC1L, ITM2C, HSPA1A
Cellular Component	blood microparticle	14	2.583025	0.00003000	FGB, FGA, ITIH4, PRSS1, SERPINF2, AFM, HBB, HBA2, PLG, HBD, HBA1, ACTG2, ALB, HSPA1A
Cellular Component	hemoglobin complex	5	0.922509	0.00023300	HBB, HBA2, HBD, HBA1

Table S9. Top 5 data for molecular functional enrichment analysis of CKD DEGs

Category	Term	Count	%	*P* Value	Genes
Molecular Function	extracellular matrix structural constituent	13	2.398523	0.000165	FGB, COL17A1, FGA, COL11A2, ADIPOQ, GP2, LAMC2, FBLN1, MUC5B, COLQ, MFAP5, CHI3L1, CCN1
Molecular Function	oligosaccharide binding	5	0.922509	0.000221	REG3A, REG1B, REG1A, ITLN1, REG3G
Molecular Function	oxygen binding	7	1.291512	0.000230	ALB, HBB, HBA2, CYP2E1, HBD, HBA1
Molecular Function	heparin binding	14	2.583025	0.000360	CXCL6, LRPAP1, MMP7, PCOLCE2, LAMC2, COLQ, CCDC80, ADAMTSL5, GPNMB, ZNF207, SAA1, SOST, CCN1, LTF
Molecular Function	oxygen transporter activity	5	0.922509	0.000530	HBB, HBA2, HBD, HBA1

Table S10. Top 5 data of KEGG pathway enrichment analysis of CKD DEGs

Category	Term	Count	%	*P* Value	Genes
Kyoto Encyclopedia of Genes and Genomes Pathway	Pancreatic secretion	12	2.214022	0.000025	PNLIPRP2, CELA3A, PRSS1, CPA1, CPB1, CELA2B, PLA2G1B, CTRB1, AMY1C, ATP1A4, PRSS2, PNLIP
Kyoto Encyclopedia of Genes and Genomes Pathway	Protein digestion and absorption	11	2.029520	0.000148	COL17A1, CELA3A, PRSS1, CPA1, CPB1, CELA2B, MME, CTRB1, COL11A2, ATP1A4, PRSS2
Kyoto Encyclopedia of Genes and Genomes Pathway	African trypanosomiasis	6	1.107011	0.001633	IL10, VCAM1, HBB, HBA2, HBA1, IDO2
Kyoto Encyclopedia of Genes and Genomes Pathway	Renin-angiotensin system	4	0.738007	0.016154	NLN, MME, KLK1, REN
Kyoto Encyclopedia of Genes and Genomes Pathway	Malaria	5	0.922509	0.029703	IL10, VCAM1, HBB, HBA2, HBA1

Table S11. List of CKD differential genes with the top 10 PPI network degree values

Node	ALB	MYC	IL10	FOS	TOP2A	PLG	REN	FGA	CCNA2	BUB1
Description	up	down	up	up	down	up	up	down	down	down
Degree	130	86	60	50	48	46	44	44	44	44

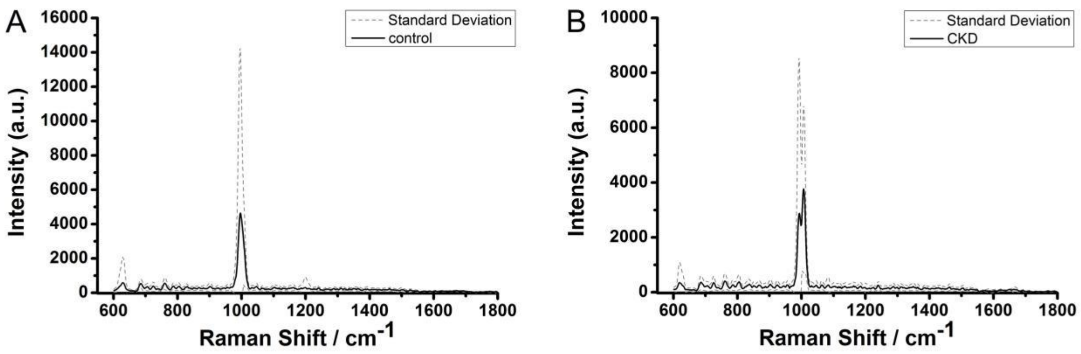

Figure S1. A: Mean Raman spectra of urine from the control group B: Mean Raman spectra of urine from the HUA combined with CKD group

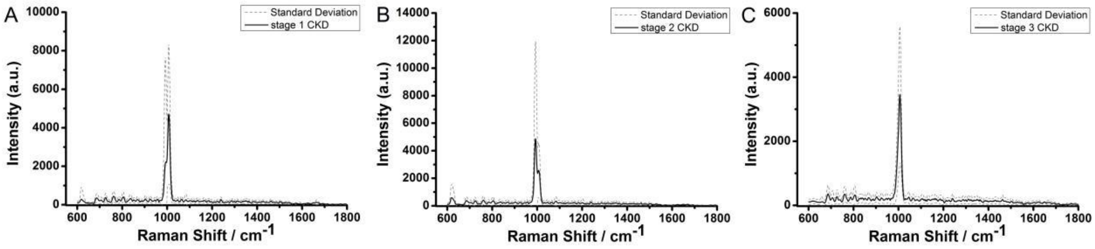

Figure S2. A: Mean Raman spectra of urine from stage 1 CKD group B: Mean Raman spectra of urine for the stage 2 CKD group C: Mean Raman spectra of urine for the stage 3 CKD group

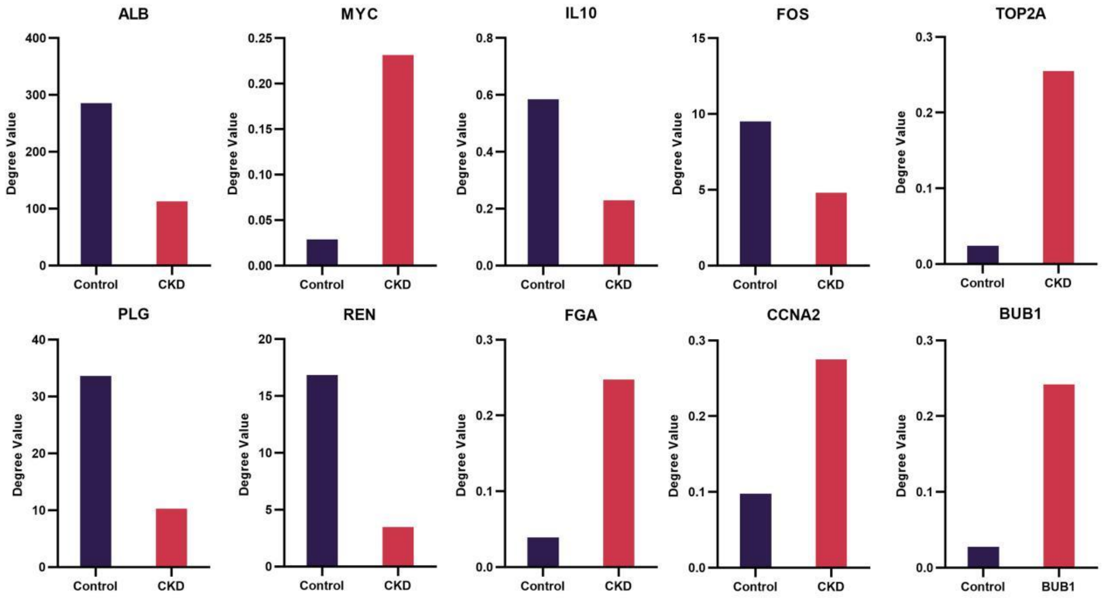

Figure S3. Intensity values of the screened key differential candidate genes: The top 10 differentially expressed genes were *ALB, MYC, IL10, FOS, TOP2A, PLG, REN, FGA, CCNA2 and BUB1*, derived from GeneC-hip database sequence number GSM1623299-GSM1623346, GSM1623352-GSM1623356, GSM1623347-GSM1623351 and GSM1623357-GSM1623359.

References

(1) Lin, J., Huang, Z., Lin, X., Wu, Q., Quan, K., Cheng, Y., Zheng, M., Xu, J., Dai, Y., Qiu, H., Lin, D., and Feng, S. (2020). Rapid and label-free urine test based on surface-enhanced Raman spectroscopy for the non-invasive detection of colorectal cancer at different stages. Biomed. Opt. Express 11, 7109–7119. doi: 10.1364/BOE.406097

(2) González-Solís, J.L., Villafan-Bernal, J.R., Martínez-Zérega, B.E., and Sánchez-Enríquez, S. (2018). Type 2 diabetes detection based on serum sample Raman spectroscopy. Lasers Med. Sci. 33, 1791–1797. doi: 10.1007/s10103-018-2543-4

(3) Chen, C., Yang, L., Li, H., Chen, F., Chen, C., Gao, R., Lv, X.Y., and Tang, J. (2020). Raman spectroscopy combined with multiple algorithms for analysis and rapid screening of chronic renal failure. Photodiag. Photodyn. Ther. 30, 101792. doi: 10.1016/j.pdpdt.2020.101792

(4) Moreira, L.P., Silveira Jr, L., da Silva, A.G., Fernandes, A.B., Pacheco, M.T.T., and Rocco, D.D.F.M. (2017). Raman spectroscopy applied to identify metabolites in urine of physically active subjects. J. Photochem. Photobiol. B Biol. 176, 92–99. doi: 10.1016/j.jphotobiol.2017.09.019

(5) Moreira, L.P., Silveira Jr, L., Pacheco, M.T.T., da Silva, A.G., and Rocco, D.D.F.M. (2018). Detecting urine metabolites related to training performance in swimming athletes by means of Raman spectroscopy and principal component analysis. J. Photochem. Photobiol. B Biol. 185, 223–234. doi: 10.1016/j.jphotobiol.2018.06.013
